# Intravenous thrombolysis for acute ischemic stroke in the extended time window of 4.5–24 h: a systematic review and network meta-analysis of randomized controlled trials

**DOI:** 10.3389/fneur.2026.1845353

**Published:** 2026-05-29

**Authors:** Xiaoqi Yu, Xiuye Sun, Fang Yang

**Affiliations:** 1 Second Clinical College, Liaoning University of Traditional Chinese Medicine, Shenyang, China; 2 Department of Rehabilitation, The Second Affiliated Hospital of Liaoning University of Traditional Chinese Medicine, Shenyang, China

**Keywords:** alteplase, intravenous thrombolysis, ischemic stroke, JX10, tenecteplase

## Abstract

**Objective:**

This study aims to systematically evaluate the efficacy and safety of different intravenous thrombolytic agents in patients with acute ischemic stroke (IS) who present between 4.5 and 24 h after symptom onset. A network meta-analysis was conducted to compare the available treatments and provide evidence to support clinical decision-making and inform updates to clinical guidelines.

**Methods:**

A systematic search was conducted in PubMed, Embase, the Cochrane Library, and Web of Science, covering the period from the inception of each database to February 1, 2026. Randomized controlled trials (RCTs) comparing different intravenous thrombolytic regimens within the extended time window were included. Primary outcomes included a modified Rankin Scale score of 0–1 at 90 days, mRS 0–2 at 90 days, improvement in the National Institutes of Health Stroke Scale at 24 h, reperfusion at 24 h, symptomatic intracranial hemorrhage at 36 h, and mortality at 90 days. A network meta-analysis using a frequentist random-effects model was conducted to report relative risks and 95% confidence intervals. Interventions were ranked using the cumulative ranked area under the curve, and the certainty of the evidence was assessed using the GRADE/CINeMA framework.

**Results:**

A total of 10 RCTs (2,409 patients) were included, involving five interventions: alteplase (rt-PA), tenaplase (TNK), JX10, standard of care (SoC), and placebo (PBO). For the primary outcome of 90-day mRS 0–2, compared with SoC, rt-PA significantly increased the proportion of functionally independent patients (RR = 1.21, 95% CI 1.06–1.38, low certainty), JX10 (RR = 1.50, 95% CI 0.87–2.58, very low certainty) and TNK (RR = 1.07, 95% CI 0.93–1.23, low certainty) showed a trend toward improvement but did not reach statistical significance. The SUCRA ranking showed that JX10 (87.7%) and rt-PA (77.0%) ranked first and second, respectively. Regarding the early surrogate endpoint of 24-h reperfusion, rt-PA (RR = 2.65, 95% CI 1.57–4.46, low certainty) was superior to SoC and ranked first in the SUCRA ranking (99.9%). Regarding safety, rt-PA significantly increased the risk of 36-h SICH compared with SoC (RR = 5.82, 95% CI 1.47–22.95, low certainty), while JX10 had the highest risk of bleeding (SUCRA 82.1%). There were no statistically significant differences in 90-day mortality rates among the interventions. The certainty of the evidence was predominantly low to very low, primarily limited by risk of bias, imprecision, and indirectness.

**Conclusion:**

In patients with acute IS within 4.5–24 h of onset, rt-PA significantly improves functional independence at 90 days and 24-h reperfusion, but increases the risk of symptomatic intracranial hemorrhage. The ranking analysis suggested JX10 might have a favorable functional outcome profile, but this finding is derived from a single, small-sample, dose-exploratory study *n* = 90 with no direct comparative data against other thrombolytics. Consequently, the evidence for JX10 is considered hypothesis-generating only, and its clinical efficacy and safety remain unestablished. Given the high risk of hemorrhage, its clinical application is not yet supported. The therapeutic advantages of TNK have not been fully demonstrated, though its safety profile is relatively superior. Clinical decision-making requires individualized consideration based on imaging screening, bleeding risk assessment, and patient preference. There is an urgent need for larger-scale, rigorously designed head-to-head RCTs to provide higher-quality evidence for expanding the time window for thrombolytic therapy.

**Systematic trial registration:**

https://www.crd.york.ac.uk/PROSPERO/view/CRD420261350208, identifier: CRD420261350208

## 1 Introduction

Ischemic stroke (IS) refers to a clinical syndrome characterized by impaired blood supply to brain tissue due to vascular causes such as atherosclerosis of large arteries, cardioembolism, or small-vessel occlusion, resulting in localized cerebral ischemia and hypoxic necrosis, and subsequently leading to corresponding neurological deficits. It accounts for approximately 87% of all stroke cases (1). Stroke has become one of the neurological conditions with the heaviest disease burden worldwide; in 2023, stroke-related disability-adjusted life years (DALYs) reached 157 million, ranking second among the global burden of noncommunicable diseases (2). According to data from the Global Burden of Disease Study 2023, stroke caused 6.79 million deaths in 2023, accounting for 10.7% of all global deaths and ranking as the second leading cause of death worldwide; IS accounted for 48.3% of all stroke-related deaths (3). From 1990 to 2021, the global incidence of stroke increased by approximately 70.20%, with the rise in IS being particularly pronounced at 87.97%, significantly higher than that of other stroke subtypes. It is projected that by 2046, the global incidence of stroke will reach 22,962,866 cases, representing a 92.5% increase from 2021 (4).

The central concept underlying the pathophysiological progression of IS lies in the existence of the “ischemic penumbra” (5). This region refers to brain tissue surrounding the infarct core where blood flow is reduced, but energy metabolism persists. Although electrical activity has ceased, ion pump function has not yet completely failed, and neurons remain viable; their survival depends heavily on the compensatory capacity of the collateral circulation and the timeliness of vascular recanalization. Therefore, in clinical practice, achieving rapid recanalization to salvage the penumbra is the central goal of all reperfusion strategies, including endovascular therapy and intravenous thrombolysis (6). Intravenous thrombolysis is currently the first-line reperfusion therapy recommended by international guidelines. It uses plasminogen activators, such as alteplase (rt-PA) and tenecteplase (TNK), to dissolve fresh blood clots and restore blood flow. The extent of its clinical benefit depends on the drug's efficacy and safety, as well as the time window from symptom onset to treatment. Traditional guidelines strictly stipulate that intravenous thrombolysis must be administered within 4.5 h of IS onset (7). In recent years, new evidence from several randomized controlled trials (RCTs), including HOPE and TIMELESS, suggests that for some patients, the treatment window can be extended to 24 h under imaging guidance, offering the possibility of reperfusion to a broader patient population. The dynamic evolution of the ischemic penumbra provides a pathophysiological basis for individualizing the treatment window (8).

It is worth noting that these trials exploring extended treatment windows exhibit significant heterogeneity in their design: some permit combined endovascular therapy, while others exclude it; control groups include either placebo or standard therapy; and the study populations encompass occlusions in both the anterior and posterior circulations, as well as in large, medium, and small vessels. This heterogeneity makes it difficult for a single RCT or traditional meta-analysis to answer the core clinical question of “which thrombolytic agent is optimal within the extended treatment window”. Network meta-analysis (NMA) can rank the relative efficacy of different interventions by integrating evidence from both direct and indirect comparisons, and can explore the effects of efficacy-modifying factors such as time-window subgroups, stroke type, and the use of combined endovascular therapy (EVT). Previous NMA studies have evaluated and compared the efficacy and safety of different intravenous thrombolysis regimens within 4.5 h of IS onset, providing systematic evidence to support clinical decision-making within this time window (9). However, comparative studies on various thrombolytic agents within the extended time window of 4.5–24 h remain relatively limited. In light of this, this study aims to use a network meta-analysis (NMA) approach to systematically evaluate and compare the safety and clinical efficacy of different intravenous thrombolytic agents used to treat IS patients within the extended time window. The findings will provide evidence-based support for clinicians in making individualized treatment decisions within the extended time window, helping more IS patients who exceed the standard time window to receive effective reperfusion therapy and further optimize treatment strategies.

## 2 Materials and methods

### 2.1 Protocol and registration

This network meta-analysis was conducted in accordance with the Extended Statement for Network Meta-Analysis (Supplementary Table S1) of the Preferred Reporting Items for Systematic Reviews and Meta-Analyses guidelines (10). To ensure transparency, reliability, and originality, the protocol for this study has been registered with the Prospective Register of Systematic Reviews under registration number CRD420261350208.

### 2.2 Search strategy

This study conducted a systematic search of the PubMed, EMBASE, Cochrane Library, and Web of Science databases. The search terms used were “Ischemic Stroke,” “Cryptogenic Embolic Stroke,” “Wake-up Stroke,” “Tissue Plasminogen Activator,” “Tissue Activator D-44,” “Plasminogen,” “Activator, Tissue-Type,” “TTPA,” “Alteplase,” “Tisokinase,” “Lysatec rt-PA,” “Tenecteplase,” “randomized controlled trial,” and “RCT” (Supplementary Table S2). The search period spanned from the inception of each database to February 1, 2026. The search method combined free-text terms with subject headings, and no language restrictions were applied.

### 2.3 Inclusion and exclusion criteria

Prior to inclusion in the RCT, studies were screened based on their titles, abstracts, and RCT identifiers. All included RCTs were double-checked by two reviewers to ensure that the data from the included RCTs were from the most recently published studies.

Inclusion criteria:

1. RCTs that clearly define the time window for IS onset as 4.5–24 h.
2. RCTs comparing the use of rt-PA, tinaplase, or JX10 alone with other treatment regimens for IS.
3. RCTs should report at least one of the following outcome measures:90-day mRS 0–1; 90d mRS 0–2; 24h NIHSS; 24h Rep; 36h Sich; 90d Death. Detailed information on all included studies is presented in Table 1.

**Table 1:** Inclusion criteria characteristics of included studies.

References	90d mRS0-1	90d mRS0-2	24h NIHSS	24h Rep	36h Sich	90d Death
Ma et al. (18)	Defined as a score of 0 or 1 on the modified Rankin scale at 90 days after IS onset	Defined as a score of 0–2 on the modified Rankin scale at 90 days after IS onset	Defined as a decrease of ≥8 points or a score of ≤ 1 on the National Institutes of Health Stroke Scale between pre-treatment and 24 h post-treatment	Defined as ≥90% reduction in the volume of the perfusion lesion (Tmax >6 s) at 24 h post-treatment	Using the ECASS/SITS-MOST criteria, sICH was defined as parenchymal hematoma type 2 within 36 h, accompanied by an NIHSS increase of ≥4 points from baseline	Defined as death within 90 days after start of assigned treatment
Zhou et al. (19)	Defined as a score of 0 or 1 on the modified Rankin scale at 90 days after IS onset	Defined as a score of 0–2 on the modified Rankin scale at 90 days after IS onset	Defined as a decrease of ≥8 points or a score of ≤ 1 on the National Institutes of Health Stroke Scale between pre-treatment and 24 h post-treatment	NA	Using the ECASS criteria, sICH was defined as any intracranial hemorrhage within 36 h that was judged to be the predominant cause of neurological deterioration, with an NIHSS increase of ≥4 points from baseline or leading to death	Defined as death within 90 days after start of assigned treatment
Yan et al. (20)	Defined as a score of 0 or 1 on the modified Rankin scale at 90 days after IS onset	Defined as a score of 0–2 on the modified Rankin scale at 90 days after IS onset	Defined as a decrease of ≥8 points or a score of ≤ 1 on the National Institutes of Health Stroke Scale between pre-treatment and 24 h post-treatment	NA	Using the ECASS criteria, sICH was defined as any intracranial hemorrhage within 36 h that was judged to be the predominant cause of neurological deterioration, with an NIHSS increase of ≥4 points from baseline or leading to death	Defined as death within 90 days after start of assigned treatment
Yogendrakumar et al. (21)	Defined as a score of 0 or 1 on the modified Rankin scale at 90 days after IS onset	Defined as a score of 0–2 on the modified Rankin scale at 90 days after IS onset	Defined as a decrease of ≥8 points or a score of ≤ 1 on the National Institutes of Health Stroke Scale between pre-treatment and 24 h post-treatment	Defined as >90% reduction in the volume of the perfusion lesion (Tmax >6 s) at 24 h post-treatment	NA	Defined as death within 90 days after start of assigned treatment
Xiong et al. (22)	Defined as a score of 0 or 1 on the modified Rankin scale at 90 days after IS onset	Defined as a score of 0–2 on the modified Rankin scale at 90 days after IS onset	NA	Defined as >90% reduction in the volume of the perfusion lesion (Tmax >6 s) at 24 h post-treatment	Using the ECASS criteria, sICH was defined as any intracranial hemorrhage within 36 h that was associated with clinical deterioration (NIHSS increase of ≥4 points from baseline) or leading to death, and identified as the predominant cause of neurological deterioration	Defined as death within 90 days after start of assigned treatment
Albers et al. (23)	NA	Defined as a score of 0–2 on the modified Rankin scale at 90 days after IS onset	NA	Defined as >90% reduction in the volume of the perfusion lesion (Tmax >6 s) at 24 h post-treatment	Defined as any intracranial bleeding within 36 h after administration of tenecteplase or placebo that caused an increase of at least 4 points on the NIHSS score from the most recent assessment	Defined as death within 90 days after start of assigned treatment
Cheng et al. (24)	Defined as a score of 0 or 1 on the modified Rankin scale at 90 days after IS onset	Defined as a score of 0–2 on the modified Rankin scale at 90 days after IS onset	NA	NA	NA	NA
Niizuma et al. (17)	Defined as a score of 0 or 1 on the modified Rankin scale at 90 days after IS onset	Defined as a score of 0–2 on the modified Rankin scale at 90 days after IS onset	NA	NA	Using a modified ECASS criteria, sICH was defined as parenchymal hematoma type 1 or 2 within 24 h accompanied by an NIHSS increase of ≥4 points from baseline	NA
Ringleb et al. (25)	Defined as a score of 0 or 1 on the modified Rankin scale at 90 days after IS onset	Defined as a score of 0–2 on the modified Rankin scale at 90 days after IS onset	NA	NA	NA	NA
Wang et al. (26)	Defined as a score of 0 or 1 on the modified Rankin scale at 90 days after IS onset	Defined as a score of 0–2 on the modified Rankin scale at 90 days after IS onset	NA	NA	NA	NA

Exclusion criteria:

1. RCTs involving patients with acute IS who have imaging evidence supporting intravenous thrombolysis but unknown time of onset.
2. RCTs with ambiguous or poorly defined outcome measures.
3. Non-randomized studies (e.g., cohort studies, case-control studies).
4. Reviews, meta-analyses, case reports, or conference abstracts.

### 2.4 Data extraction

The researchers independently extracted data from RCTs in accordance with the Preferred Reporting Items for Systematic Reviews and Meta-Analyses guidelines. Any discrepancies were resolved through discussion with the second author. From each article, the following information was extracted: RCT designation, first author, year of publication, sample size, patient age, gender and geographic distribution, follow-up duration, intervention protocols for the treatment and control groups, whether patients received endovascular treatment, and stroke circulation type. For dichotomous outcomes, the number of events and total number of cases in each group were extracted.

This study conducted a quality assessment using ROB2, a tool based on the evidence-based principles of systematic reviews and meta-analyses. It systematically evaluated potential sources of bias in the studies across five distinct domains, including the randomization process, deviation from the intended intervention, missing outcome data, outcome measurement, and selective reporting. Each domain and the overall assessment were rated as low risk, some concerns, or high risk (11).

### 2.5 Statistical analysis

This study conducted a network meta-analysis using Stata 17.0 MP (StataCorp LLC, College Station, TX, USA). For dichotomous outcomes, the relative risk (RR) and its 95% confidence interval were reported. For multi-arm trials, pairwise comparisons were generated using the augment format, and the within-study correlation structure was retained in the estimates to avoid underestimation of standard errors due to the reuse of control groups. For binary outcomes, if a study had zero events or zero non-events, continuity corrections were applied to all treatment arms within that study *r* = *r*+1, *n* = *n*+1 prior to calculating the effect size to avoid infinite RR estimates. The primary analysis was conducted using a random-effects model under the assumption of homogeneity, and the between-study variance (τ^2^) was estimated using the REML method. If the network contains a closed-loop structure, global inconsistency is assessed using a global inconsistency test, while local inconsistency is evaluated using the node-splitting method; a *p*-value < 0.05 is considered an indicator of potential inconsistency. Closed-loop consistency is assessed using the loop inconsistency factor (IF); if the 95% confidence interval of the IF includes 0, this indicates that no statistical evidence was found to support inconsistency between direct and indirect evidence. A network diagram was created to visualize the network's structure. In the network diagram, node size is proportional to the total sample size for each treatment, and the thickness of the connecting lines represents the number of studies directly comparing the interventions. To rank the interventions, we applied multiple ranking metrics, including the cumulative area under the ranking curve (SUCRA), the probability of the best treatment (PreBest), and the average rank, to enhance the robustness and interpretability of the results. Publication bias and small-sample effects were assessed using funnel plots adjusted for comparisons. Robustness testing was conducted using leave-one-out sensitivity analysis, in which the random-effects consistency model was repeated after sequentially excluding individual studies, and the direction and magnitude of the pooled effects were compared. Furthermore, univariate network meta-regression was used to explore the influence of study-level covariates on treatment effects, reporting regression coefficients, 95% confidence intervals, and Wald test *p*-values; a *p*-value < 0.05 was considered statistical evidence suggesting a moderating effect of the covariate.

### 2.6 GRADE Classification

This study assessed the certainty of evidence from the network meta-analysis results using the GRADE framework and Confidence in Network Meta-analysis (CINeMA). RCTs were initially assigned a “high certainty” rating. The certainty of the evidence was assessed comprehensively across six domains: within-study bias, indirectness, imprecision, heterogeneity, inconsistency, and between-study bias (publication bias/small-sample effect). In-study bias was assessed domain-by-domain using the RoB 2.0 tool, and the risk of bias was weighted and aggregated at the comparison level using the CINeMA contribution matrix based on its contribution to the network estimate. Indirectness was assessed based on the assumptions of transitivity and commutativity. Potential effect modifiers (such as baseline severity, intervention intensity, and follow-up duration) were predefined to compare the consistency of direct and indirect evidence across populations, interventions, comparators, and outcome measures. Imprecision is assessed using a predefined minimum clinically important difference (MID) as the threshold: for binary outcomes, an RR of 1.25 is used as the threshold for clinically significant effects, and the assessment is based on whether the 95% confidence interval crosses the null line and meets the aforementioned threshold. Heterogeneity was primarily assessed based on the τ^2^ estimated by the random-effects model and the position of the predicted interval relative to the MID. For networks with a closed loop, inconsistency was comprehensively evaluated using CINeMA's built-in direct-indirect consistency assessment (based on node splitting/SIDE or the design-by-treatment method). Study-to-study bias is assessed by considering trial registration and gray literature searches, and by referencing small-sample effects indicated by comparative-adjusted funnel plots. Each domain is graded as “no concerns,” “some concerns,” or “serious concerns,” and downgraded according to GRADE principles: “some concerns” results in a one-level downgrade, and “serious concerns” results in a two-level downgrade. The certainty of the evidence is ultimately classified as high, moderate, low, or very low.

## 3 Results

### 3.1 Study selection

**Figure 1:**
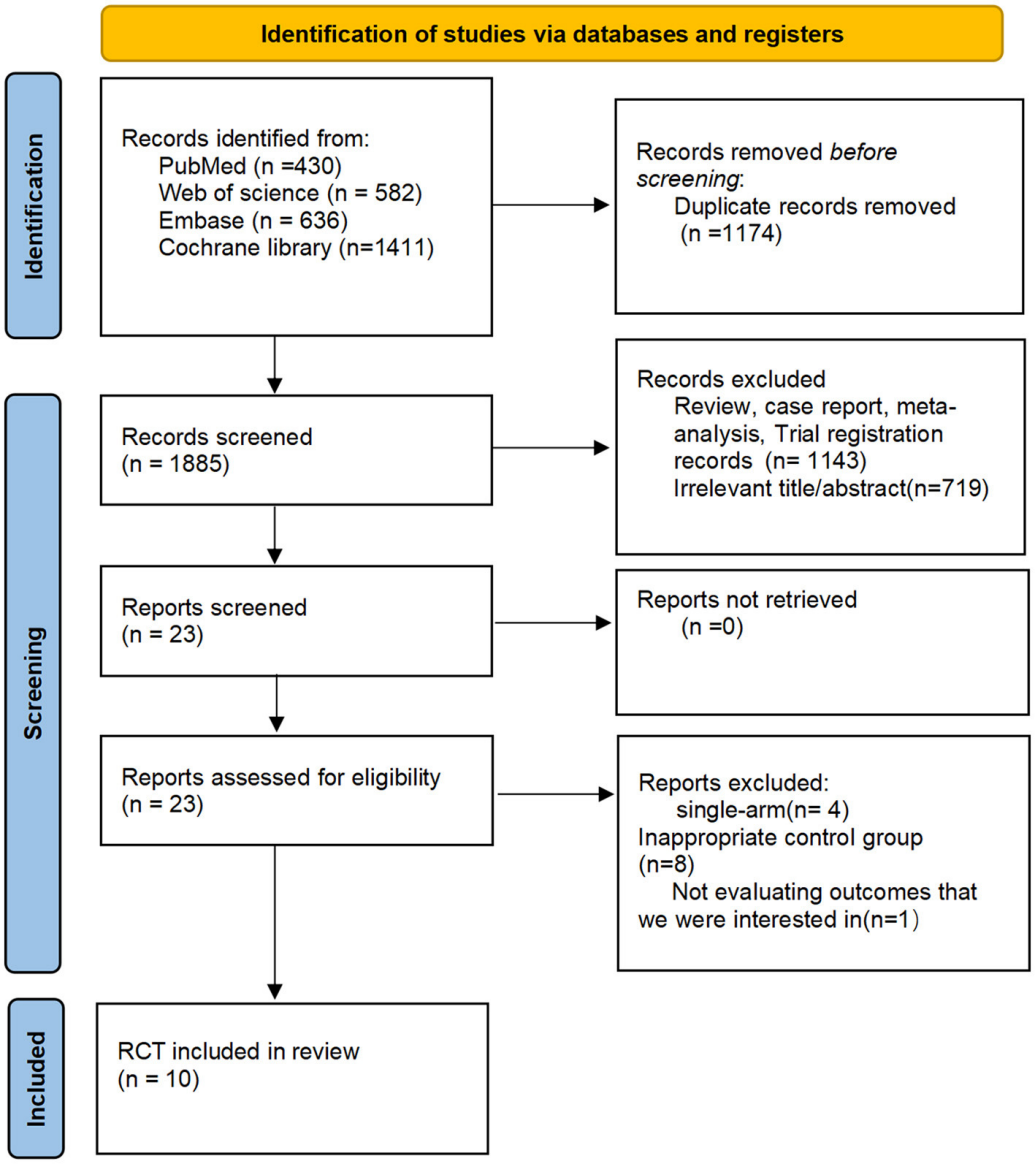
Literature search and study selection flow diagram. RCT, randomized controlled trial.

In our initial literature search, we identified a total of 3,059 records in the databases. After screening the abstracts to exclude duplicate and irrelevant articles, 23 studies were deemed eligible for full-text review; ultimately, 10 studies met our inclusion and exclusion criteria (Figure 1).

This study enrolled a total of 2,409 patients who received one of the following five interventions: tenecteplase (TNK), recombinant tissue-type plasminogen activator (rt-PA), JX10, standard of care (SoC), or placebo (PBO). The drug doses for each experimental group are as follows: TNK 0.25 mg/kg, rt-PA 0.9 mg/kg, JX10 (6 mg/kg). The JX10 intravenous thrombolysis RCT was a dose-finding trial that included three dose groups: 1, 3, and 6 mg/kg. To facilitate presentation in the network meta-analysis, the 6 mg/kg group, which had the largest sample size and accounted for 53.8% of the total experimental group is used as the representative group here, as its efficacy was similar to that of the 3 mg/kg group. The study spanned multiple data centers worldwide, including Asia (7), Europe (2), Oceania (2), and North America (1). The total sample size was moderate, consisting primarily of middle-aged and older patients (with an average age ranging from 62 to 78 years), and the gender distribution was predominantly male. The follow-up period after intervention was consistent across all studies, at 90 days. Detailed information on all included studies is presented in Table 2.

**Table 2:** Baseline characteristics of included studies.

Reference	Randomizedcontrolled trial	Trial registration number	Time of onset	Stroke classification by circulatory territory	Type of EVT	Median NIHSS	Intervention	Control	Follow-up	No. of patients	Age, years	Gender	Continent
Ma et al. (18)	EXTEND	NCT00887328 NCT01508393	4.5–9 h	Anterior circulation stroke Posterior circulation stroke	No EVT	TL: 12 (8–17) NO-TL: 10 (6–16.5)	rt-PA 0.9 mg/kg	Placebo	90 days	225 TL: 113 NO-TL: 112	TL: 73.7 ± 12.7 NO-TL: 71.0 ± 12.7	TL: NO-Male 52.2% TL: NO-Female 47.8% NO-TL: Male 58.9% NO-TL: Female 41.1%	Oceania Europe Asia
Zhou et al. (19)	HOPE	NCT04879615	4.5–24 h	Anterior circulation stroke Posterior circulation stroke	Rescue EVT	TL: 10 (6–15) NO-TL: 10 (6–14)	rt-PA 0.9 mg/kg	Antiplatelet therapy and other supportive care	90 days	372 TL: 186 NO-TL: 186	TL: 72 (62–80) NO-TL: 73 (65–80)	TL: NO-Male 52.2% TL: NO-Female 47.8% NO-TL: Male 58.9% NO-TL: Female 41.1%	Asia
Yan et al. (20)	EXPECTS	NCT05429476	4.5–24 h	Posterior circulation stroke	Rescue EVT	TL: 3 (2–6) NO-TL: 3 (1–6)	rt-PA 0.9 mg/kg	Antiplatelet therapy and other supportive care	90 days	234 TL: 117 NO-TL: 117	TL: 64 (57–76) NO-TL: 63 (55–74)	TL: NO-Male 64.1% TL: NO-Female 35.9% NO-TL: Male 66.7% NO-TL: Female 33.3%	Asia
Yogendrakumar et al. (21)	ETERNAL-LVO	NCT04454788	4.5–24 h	Anterior Circulation stroke	EVT	TL: 13 (7–19) NO-TL: 14 (7–18)	TNK 0.25 mg/kg	Standard medical therapy (excluding alteplase)	90 days	99 TL: 50 NO-TL: 49	TL: 73 (64–81) NO-TL: 72 (62–79)	TL: NO-Male 55% TL: NO-Female 45% NO-TL: Male 59% NO-TL: Female 41%	Oceania
Xiong et al. (22)	TRACE-III	NCT05141305	4.5–24 h	Anterior Circulation stroke	Rescue EVT	TL: 11 (7–15) NO-TL: 10 (7–14)	TNK 0.25 mg/kg	Antiplatelet therapy and other supportive care	90 days	516 TL: 264 NO-TL: 252	TL: 67 (58–75) NO-TL: 68 (59–76)	TL: NO-Male 69.3% TL: NO-Female 30.7% NO-TL: Male 66.3% NO-TL: Female 33.7%	Asia
Albers et al. (23)	TIMELESS	NCT03785678	4.5–24 h	Anterior circulation stroke	EVT	TL: 12 (8–17) NO-TL: 12 (8–18)	TNK 0.25 mg/kg	Placebo	90 days	458 TL: 228 NO-TL: 230	TL: 72 (62–79) NO-TL: 73 (63–82)	TL: NO-Male 53.5% TL: NO-Female 46.5% NO-TL: Male 53.5% NO-TL: Female 46.5%	North America
Cheng et al. (24)	CHABLIS-TII	NCT04516993	4.5–24 h	Anterior circulation stroke	EVT	TL: 9 (5–14) NO-TL: 9 (6–16)	TNK 0.25 mg/kg	Standard medical therapy (excluding alteplase)	90 days	224 TL: 111 NO-TL: 113	TL: 64.2 ± 10.4 NO-TL: 63.6 ± 11.0	TL: NO-Male 72.1% TL: NO-Female 27.9% NO-TL: Male 70.8% NO-TL: Female 29.2%	Asia
Niizuma et al. (17)	NA	jRCT2080223786	4.5–12 h	Anterior circulation stroke	No EVT	TL: 8 (6–21) NO-TL: 8 (6–22)	JX10 6 mg/kg	Placebo	90 days	90 TL: 52 NO-TL: 38	TL: 76.5 (42–87) NO-TL: 75 (34–85)	TL: NO-Male 65.4% TL: NO-Female 34.6% NO-TL: Male 73.7% NO-TL: Female 26.3%	Asia
Ringleb et al. (25)	ECASS-4	ISRCTN71616222	4.5–9 h	Anterior circulation stroke	No EVT	10.6	rt-PA 0.9 mg/kg	Placebo	90 days	119 TL: 61 NO-TL: 58	78	Male 56.3% Female 43.7%	Europe
Wang et al. (26)	ROSE-TNK	NCT04752631	4.5–24 h	Anterior circulation stroke Posterior circulation stroke	No EVT	TL: 7.5 (6–10.75) NO-TL: 7 (6–8.75)	TNK 0.25 mg/kg	Antiplatelet therapy and other supportive care	90 days	80 TL: 40 NO-TL: 40	TL: 62.68 ± 8.87 NO-TL: 62.80 ± 8.56	TL: NO-Male 77.5% TL: NO-Female 22.5% NO-TL: Male 65.0% NO-TL: Female 35.0%	Asia

### 3.2 Evaluation of study quality

**Figure 2:**
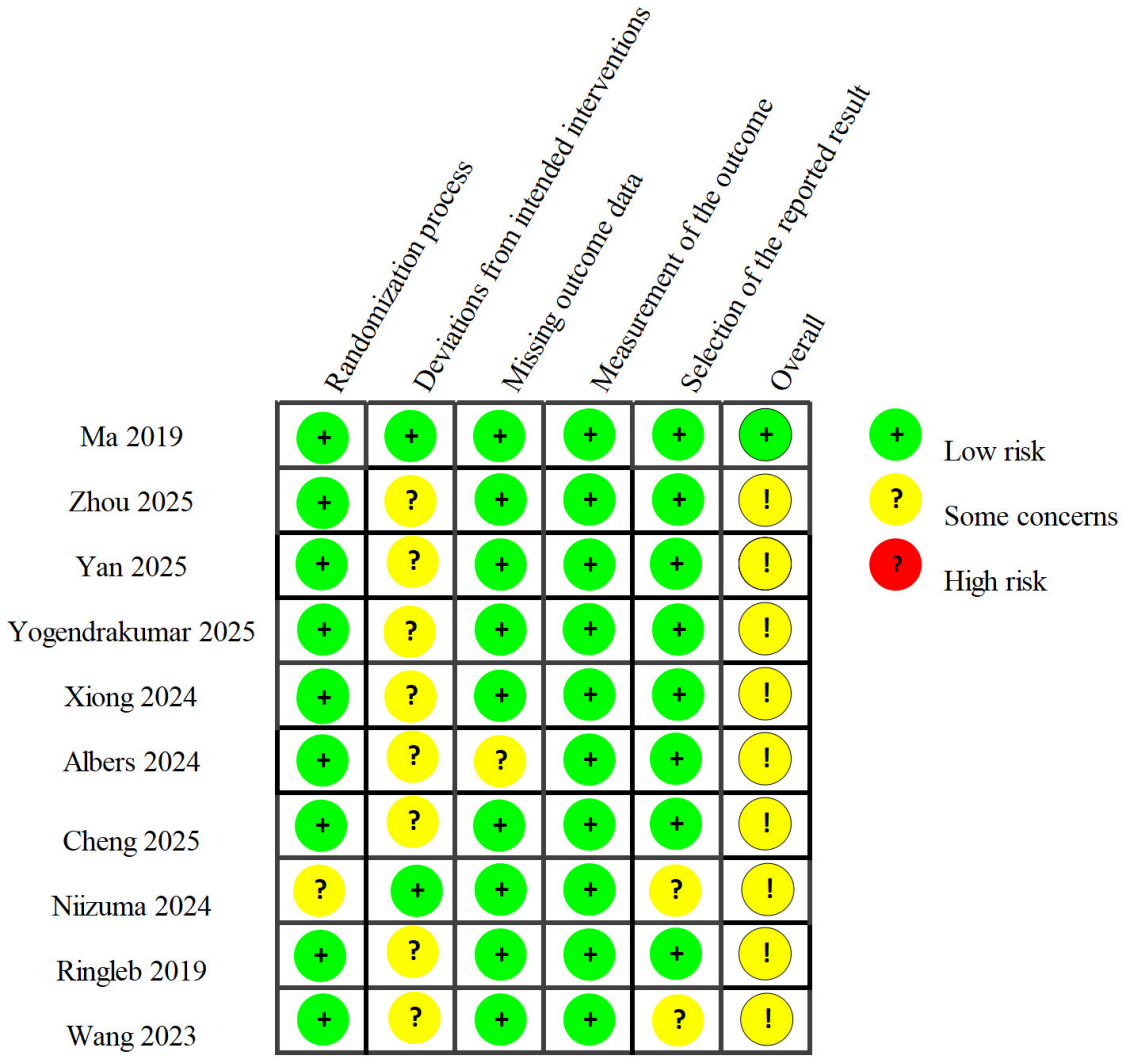
RoB2 quality assessment of studies.

This study used the RoB2 tool to assess the risk of bias in the 10 included RCTs. Overall, one study was judged to be at low risk, nine studies were classified as having “some concerns,” and no studies were found to be at high risk overall, suggesting that the overall quality of the included evidence is high. Regarding the randomization process, nine studies were assessed as having a low risk of bias. Two studies were rated as having a low risk of deviation from the intended intervention. In the assessment of missing outcome data, nine studies were classified as having a low risk. All 10 studies were rated as having a low risk of measurement bias, and eight studies were considered to have a low risk of selection bias (Figure 2).

### 3.3 Homogeneity and heterogeneity

For the outcomes 90d mRS 0–2, 36h Sich, and 90d Death, closed loops were present in the networks for each outcome; therefore, global inconsistency tests were conducted. The results showed P-values of 0.6131, 0.3414, and 0.5450, respectively, all of which were greater than 0.05, indicating no significant global inconsistency (Supplementary Table S3). Further evaluation of local inconsistency using the node-splitting method showed that the P-values for all comparison nodes were also greater than 0.05 (Supplementary Tables S4–S6). Additionally, consistency between direct and indirect evidence was examined via loop inconsistency analysis. The results showed that the confidence intervals for all loop inconsistency factors spanned 0, indicating good overall consistency of the network model (Supplementary Figures S1–S3). Therefore, the primary analysis employed a consistency model for the network meta-analysis under the consistency assumption.

Since the outcome measures of 90-day mRS 0–1, 24-h NIHSS, and 24-h Rep did not form a closed loop, it was not possible to conduct global heterogeneity tests, local heterogeneity tests, or loop-specific heterogeneity analyses. Therefore, a random-effects model was directly applied to perform a network meta-analysis under the assumption of homogeneity, and the validity of the transitivity assumption was assessed by comparing key clinical characteristics. Based on the baseline information in Table 2, the studies showed minimal differences in terms of gender, age, time window, and follow-up duration, and were generally comparable. In the assessment of evidence quality, given the lack of a closed loop and uncertainties regarding transmissibility, we downgraded the results for this outcome measure in the indirectness dimension; therefore, this conclusion should be interpreted with caution.

Overall, heterogeneity was low across all direct comparisons included in the analysis. The τ values for 24-h Rep, 36-h Sich, and 90-day Death were all below 0.001, indicating that heterogeneity among studies was negligible (Supplementary Table S7).

### 3.4 Effectiveness indicators

#### 3.4.1 90d mRS0-1

**Figure 3:**
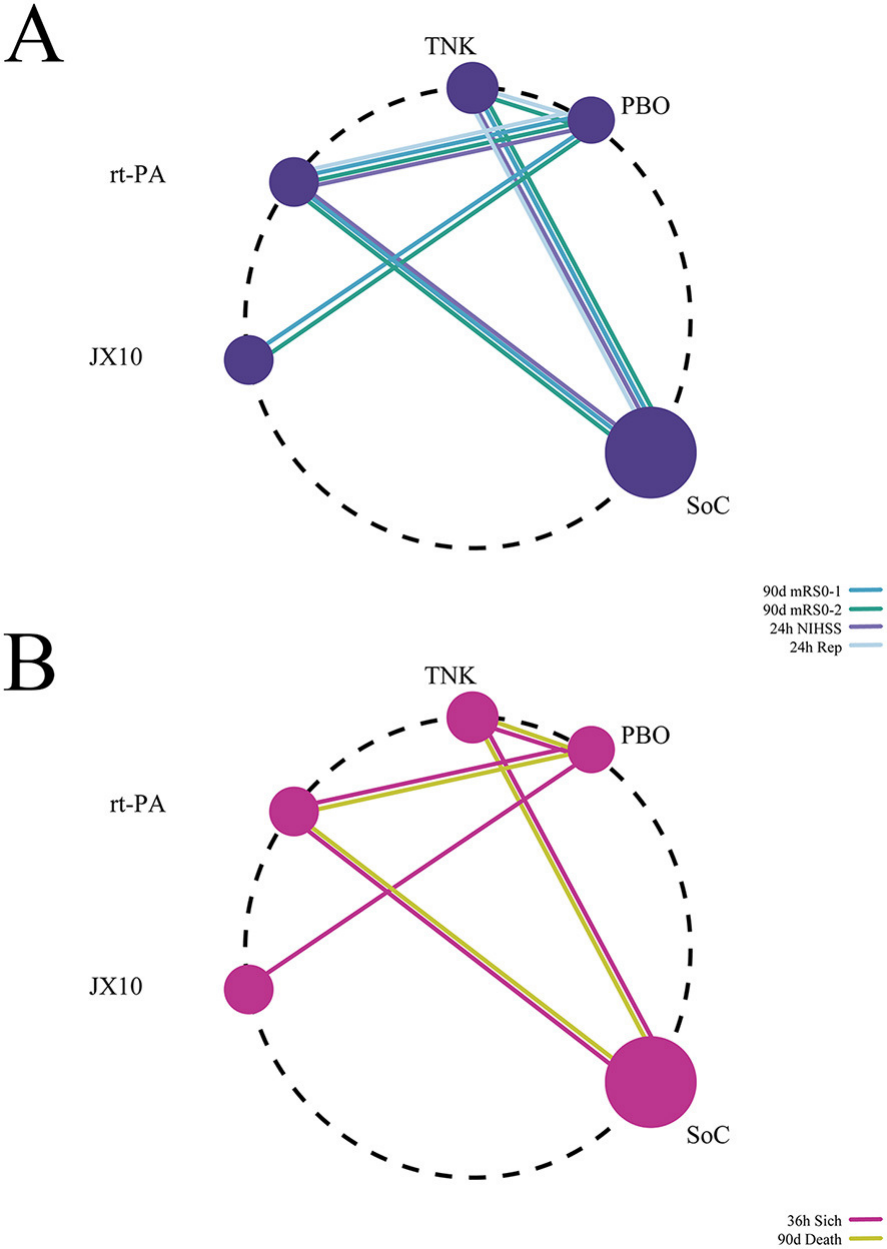
Network plots for Ischemic Stroke. **(A)** Efficacy outcomes: 90d mRS0-1, 90d mRS0-2, 24h NIHSS, 24h Rep; **(B)** Safety outcomes: 36h Sich, 90d Death. 90d mRS0-1, 90-day modified Rankin Scale 0–1; 90d mRS0-2, 90-day modified Rankin Scale 0–2; 24h NIHSS, 24-h National Institutes of Health Stroke Scale; 24h Rep, 24-h reperfusion; 36h Sich, 36-h symptomatic intracranial hemorrhage; 90d Death, 90-day death.

This analysis summarizes data from nine studies on 90-day mRS 0–1 outcomes, which evaluated the efficacy of five interventions in 1,954 patients with IS (Figure 3A).

**Figure 4:**
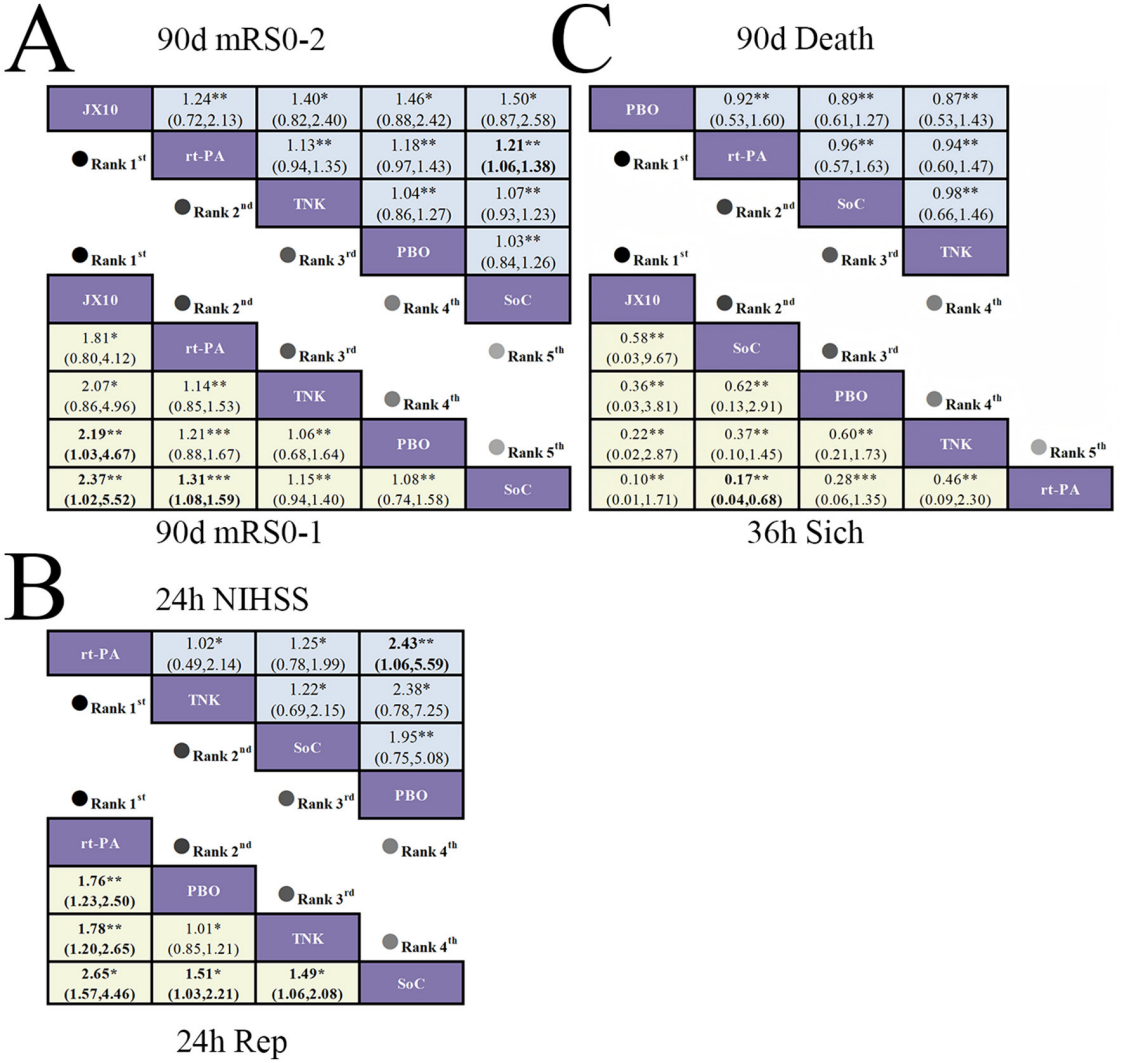
League table from the network meta-analysis. **(A)** RR and 95% CIs are presented for 90d mRS 0–1 (lower-left triangle) and 90d mRS 0–2 (upper-right triangle); **(B)** RR and 95% CIs are presented for 24h Rep (lower-left triangle) and 24h NIHSS (upper-right triangle); **(C)** RR and 95% CIs are presented for 36h Sich (lower-left triangle) and 90d Death (upper-right triangle). Symbols indicate the certainty of evidence according to GRADE classification: ^***^ = moderate; ^**^ = low; ^*^ = very low.

Moderate-to-low certainty evidence suggests that, compared with standard of care (SoC), rt-PA (RR = 1.31, 95% CI 1.08–1.59) and JX10 (RR = 2.37, 95% CI 1.02–5.52) significantly increase the proportion of patients achieving an mRS score of 0–1 at 90 days. Compared with SoC, TNK (RR = 1.15, 95% CI 0.94–1.40) showed a trend toward increasing the incidence of mRS 0–1 at 90 days, but the difference was not statistically significant; the evidence grade was low (Figure 4A).

In the SUCRA ranking, JX10 showed the highest point estimate (95.3%), but this result must be interpreted with extreme caution given that it derives from a single small-sample study with no head-to-head comparisons against other active treatments. The ranking should not be interpreted as evidence of superior efficacy. Probability analysis also indicated that JX10 was the most likely to be the most effective intervention (a posteriori probability of 90.8%), followed by rt-PA (6.9%) and TNK (2.2%). Furthermore, the average rank analysis further corroborates the relative advantage of JX10: JX10 ranked first with the lowest average rank of 1.2, followed closely by rt-PA (2.2) and TNK (3.3) (Supplementary Table S8).

#### 3.4.2 90d mRS0-2

This analysis summarizes data from 10 studies on 90-day mRS 0–2 outcomes, which evaluated the efficacy of five interventions in 2,409 patients with IS (Figure 3A).

Low-certainty evidence suggests that, compared with standard of care (SoC), rt-PA (*RR* = 1.21, 95% CI 1.06–1.38) significantly increases the incidence of mRS 0–2 at 90 days. Compared with SoC, placebo (*RR* = 1.03, 95% CI 0.84–1.26), TNK (*RR* = 1.07, 95% CI 0.93–1.23), and JX10 (*RR* = 1.50, 95% CI 0.87–2.58) showed a trend toward increasing the incidence of 90-day mRS 0–2, but the differences were not statistically significant, and the evidence quality ranged from low to very low (Figure 4A).

According to the SUCRA results, JX10 ranked first with a score of 87.7%, followed closely by rt-PA at 77%, demonstrating the relative advantages of both in terms of overall efficacy, while TNK ranked third at 42.4%. Probability analysis also showed that JX10 was the most likely to be the most effective intervention (a posteriori probability of 77.2%), followed by rt-PA (20.9%) and TNK (1.6%). Furthermore, the average rank analysis further corroborates the relative advantage of JX10: JX10 ranked first with the lowest average rank of 1.5, followed by rt-PA (1.9) and TNK (3.3) (Supplementary Table S9).

#### 3.4.3 24h NIHSS

This analysis summarizes data from four studies on the 24-h NIHSS, which evaluated the efficacy of four interventions in 1,072 patients with IS (Figure 3A).

Compared with SoC, TNK (RR = 1.22, 95% CI 0.69–2.15) and rt-PA (RR = 1.25, 95% CI 0.78–1.99) showed a trend toward an increased incidence of NIHSS at 24 h, but the differences were not statistically significant, and the level of evidence was very low (Figure 4B).

According to the SUCRA results, rt-PA ranked first with a score of 77.2%, followed closely by TNK at 73.1%, demonstrating the relative advantages of both in terms of overall efficacy. Probability analysis indicates that rt-PA is most likely to be the most effective intervention (*a priori* probability of 48.2%), followed by TNK (46.5%). Furthermore, the average rank analysis further confirms the relative advantage of rt-PA: rt-PA ranked first with the lowest average rank of 1.7, followed closely by TNK (1.8) (Supplementary Table S10).

#### 3.4.4 24h Rep

The 24h Rep synthesizes data from four studies that evaluated the efficacy of four interventions in 1,270 patients with IS (Figure 3A).

Very low-certainty evidence suggests that, compared with standard of care (SoC), rt-PA (*RR* = 2.65, 95% CI 1.57–4.46), placebo (*RR* = 1.51, 95% CI 1.03–2.21), and TNK (*RR* = 1.49, 95% CI 1.06–2.08) significantly increase the incidence of recurrent stroke within 24 h compared with SoC (Figure 4B).

According to the SUCRA results, rt-PA ranked first with a score of 99.9%, demonstrating its superiority in overall efficacy, while TNK ranked third with a score of 47.8%. Probability analysis also showed that rt-PA was the most likely to be the most effective intervention (a posteriori probability of 99.6%). Furthermore, the average ranking analysis further confirmed the superiority of rt-PA: rt-PA ranked first with the lowest average ranking of 1.0, while PBO (2.5) and TNK (2.6) ranked second and third, respectively (Supplementary Table S11).

### 3.5 Safety indicators

#### 3.5.1 36-h Sich

The 36h Sich meta-analysis synthesized data from six studies that evaluated the efficacy of five interventions in 1,862 patients with IS (Figure 3B).

Low-certainty evidence suggests that, compared with rt-PA, SoC (*RR* = 0.17, 95% CI 0.04–0.68) reduces the incidence of symptomatic bleeding within 36 h. Compared with SoC, JX10 (*RR* = 0.58, 95% CI 0.03–9.67) showed a trend toward reducing the incidence of symptomatic hemorrhage within 36 h, but the difference was not statistically significant; the evidence is of low quality (Figure 4C).

According to the SUCRA results, JX10 ranked first with a score of 82.1%, demonstrating its advantage in minimizing the risk of symptomatic bleeding in IS patients within 36 h. rt-PA ranked last with 7.4%, and TNK ranked second-to-last with 30.2%, indicating their relative disadvantages in terms of symptomatic bleeding within 36 h. Probability analysis also showed that JX10 was least likely to cause symptomatic bleeding (best predicted probability: 63.2%). Analysis of average rankings further confirmed JX10's low risk of symptomatic bleeding: JX10 ranked first with the lowest average ranking of 1.7, while rt-PA (4.7) and TNK (3.8) ranked fifth and fourth, respectively (Supplementary Table S12).

#### 3.5.2 90d Death

The analysis of 90-day mortality data from six studies evaluated the efficacy of four interventions in 1,876 patients with IS (Figure 3B).

Low-certainty evidence suggests that, compared with SoC, PBO (*RR* = 0.89, 95% CI 0.61–1.27) and rt-PA (*RR* = 0.96, 95% CI 0.57–1.63) are associated with a trend toward an increased 90-day mortality rate; however, the differences were not statistically significant, and the evidence is of low quality (Figure 4C).

According to the SUCRA results, PBO ranked first with a score of 69.1%, followed by rt-PA in second place with 52.2% and TNK in third place with 40.1%, indicating that rt-PA and TNK are relatively associated with a higher risk of death within 90 days compared to PBO. Probability analysis also showed that PBO was least likely to result in death within 90 days (best-predicted probability of 46.6%), followed by rt-PA (30%) and TNK (10.5%). Analysis of mean rankings indicated that PBO (1.9) was least likely to result in death within 90 days, followed by rt-PA (2.4) (Supplementary Table S13).

### 3.6 Meta-analysis, sensitivity analysis, and publication bias

The distribution of key baseline characteristics was generally balanced across treatment groups, with no significant differences observed. Meta-regression analysis showed that neither the type of IS circulation nor the receipt of EVT had a significant impact on treatment outcomes, supporting the validity of the transitivity hypothesis (Supplementary Tables S14–S25).

This study employed a stepwise exclusion method for sensitivity analysis to assess the impact of individual studies on the estimated network effects. The results showed that for efficacy endpoints with a larger number of included RCTs (90-day mRS 0–1 and 90-day mRS 0–2), regardless of which study was excluded, the direction of the relative effects of each intravenous thrombolytic agent compared to SoC remained consistent, and the fluctuations in the absolute effect sizes were small. the 95% confidence intervals overlapped extensively, and the assessment of statistical significance did not undergo substantial changes, suggesting that the results of the primary analysis are robust. For the safety outcome 90-day Death, no substantial changes were observed in the aforementioned sensitivity analysis, and the results were similarly robust. However, for the safety outcome 36-h SICH, the original statistical significance disappeared (or the direction of effect changed) when either the Zhou 2025 or Yan 2025 study was excluded, suggesting that these two studies had a significant impact on the network effect estimates for this outcome (Supplementary Tables S26–S29).

In this study, funnel plots were constructed for each outcome measure to assess the risk of small-sample effects and publication bias. The results showed that the scatter plots were generally symmetrical, with no obvious systematic skewness or extreme outliers observed, suggesting a low likelihood of publication bias (Supplementary Figures S4–S9).

### 3.7 Grade classification

Based on the GRADE framework and using CINeMA to assess the certainty of evidence estimated from the networks (Supplementary Figures S30–S35), the distribution of evidence certainty across the six networks 68 comparing primary and all outcomes was as follows: 0 with high certainty, five with moderate certainty, 45 with low certainty, and 18 with very low certainty. Comparisons with higher certainty of evidence were typically supported by multiple direct head-to-head trials, with confidence intervals that did not cross the null line or the MID; comparisons with lower or very low certainty of evidence were often due to sparse networks or a primary reliance on indirect evidence, accompanied by wide confidence intervals or potential small-sample effects.

## 4 Discussion

### 4.1 Key findings

For patients with acute IS presenting between 4.5 and 24 h after symptom onset, the optimal choice among intravenous thrombolytic regimens remains a clinical challenge. This network meta-analysis synthesizes direct and indirect evidence to systematically evaluate the efficacy and safety of rt-PA, TNK, and JX10 in the extended treatment window.

Based on 10 RCTs (2,409 patients), rt-PA significantly increased the proportion of patients achieving functional independence (mRS 0–2) at 90 days compared with standard therapy (moderate certainty), while JX10 showed a significant advantage in achieving good functional outcomes (mRS 0–1; low certainty). Regarding early surrogate endpoints, rt-PA was most effective in achieving 24-h reperfusion (moderate certainty). In ranking analysis, JX10 ranked first for 90-day functional outcomes, whereas rt-PA dominated for early reperfusion. For safety, rt-PA significantly increased the risk of symptomatic intracranial hemorrhage within 36 h (low certainty), and JX10 also carried a higher bleeding risk. No significant differences in 90-day mortality were observed among interventions. Notably, the evidence for JX10 derives solely from a single small-sample, dose-exploratory study; its ranking advantage should be considered highly exploratory and not confirmatory.

### 4.2 Analysis of results

These findings reveal significant differences in the benefit-risk profiles of thrombolytic strategies in the extended treatment window, offering a new evidence-based perspective for clinical decision-making, though conclusions should be interpreted cautiously given the overall low certainty of evidence.

As the standard thrombolytic agent, rt-PA activates plasmin to cleave fibrin and restore blood flow. Its significant advantage in 24-h reperfusion is consistent with its trend toward improved long-term functional outcomes, supporting its use beyond 4.5 h in patients meeting imaging criteria (e.g., presence of a salvageable penumbra). The low certainty of evidence for rt-PA's increased sICH risk reflects considerable uncertainty. Compared with the early treatment window, patients in the extended window often present with more complex collateral circulation, variable infarct core volumes, and altered blood-brain barrier integrity, making hemorrhage risk assessment more challenging.

TNK, a modified version of rt-PA, offers theoretical advantages in fibrin selectivity and half-life. However, TNK 0.25 mg/kg did not demonstrate a statistically significant advantage over standard therapy for 90-day functional outcomes. This finding is consistent with a recent systematic review and meta-analysis specifically examining TNK use beyond 4.5 h, which concluded that while TNK appears safe in the extended window, its superiority over standard care or rt-PA has not been definitively established (12). Several factors may explain this: the complex pathophysiology of extended-window patients (collateral status, thrombus composition, blood-brain barrier integrity), potential interference from concomitant endovascular therapy in some trials, and uncertainty regarding whether 0.25 mg/kg is the optimal dose in this setting.

In this network meta-analysis, the head-to-head comparison between rt-PA and TNK did not reveal significant differences in functional outcome or early reperfusion. From a pharmacological perspective, TNK possesses higher fibrin specificity, theoretically offers superior thrombolytic efficiency, and causes less systemic plasminogen consumption; therefore, its bleeding risk should be lower. However, a potential concern exists: when the time window is further extended, the thrombus in the target vessel tends to be more compact and retracted, presenting a framework rich in platelets and highly cross-linked fibrin, which may differ substantially in composition from fresh thrombi in the hyperacute phase. Whether the thrombolytic efficiency advantage of TNK can be fully retained against such older, retracted thrombi remains unclear. Moreover, TNK has a longer half-life, which facilitates single intravenous bolus administration but may also result in prolonged exposure of the already impaired blood-brain barrier to an active protease. This variable might partially offset its theoretical safety benefit. The ongoing TRAILBLAZER study (NCT07419997) is a large observational cohort study in the Beijing area directly comparing alteplase and tenecteplase, and is expected to provide real-world evidence on the effectiveness and safety under extended time windows.

The novel anti-inflammatory thrombolytic agent JX10 combines thrombolytic and anti-inflammatory effects, theoretically mitigating reperfusion injury while restoring blood flow, which may explain its high ranking in functional outcomes. However, this evidence is extremely weak, based on a single small-sample, dose-exploratory study (90 patients) lacking direct comparisons with other thrombolytics. This conclusion is corroborated by a recent comprehensive review of novel thrombolytic agents, which noted that JX10 remains in early-stage clinical investigation with an insufficient evidence base to support routine clinical use (13). Its high bleeding risk ranking also raises clinical concerns. For patients beyond the standard treatment window, pursuing functional benefits must be balanced against hemorrhage risk.

### 4.3 Implications for clinical practice and research

This analysis provides key evidence to support thrombolysis decisions in the extended time window. The results reaffirm that, with appropriate radiological screening, rt-PA is a reasonable and effective strategy beyond 4.5 h, but should be strictly limited to suitable patients with full disclosure of hemorrhage risk.

Among newer agents, TNK shows a relatively favorable safety profile, but its therapeutic advantage remains unproven. Evidence for JX10 is insufficient; it remains investigational and is not yet supported for routine clinical use. In practice, thrombolytic selection should integrate neuroimaging characteristics (penumbra extent, infarct volume), bleeding risk assessment, and patient expectations.

From a research perspective, this review identifies critical evidence gaps: most studies lack direct comparisons, and the overall certainty of evidence is low. This conclusion aligns with a recent Bayesian meta-analysis and umbrella review, which found that the evidence base for extended-window thrombolysis remains insufficient to definitively establish net clinical benefit (14). High-quality head-to-head RCTs are urgently needed, particularly direct comparisons between JX10 and standard agents, along with standardized core outcome sets to enhance evidence consistency and comparability. Real-world studies and long-term follow-up data are also essential to evaluate the benefit-risk balance across diverse clinical settings.

### 4.4 Comparison with previous studies

This study both aligns with and extends previous meta-analyses. Unlike prior NMAs (15, 16) that focused primarily on the early treatment window, we strictly limited the population to the 4.5–24 h extended window. Unlike recent paired meta-analyses comparing “thrombolysis overall” with controls, we distinguish the relative performance of individual agents. We included the most recent JX10 trials and used only the 0.25 mg/kg dose of TNK to minimize dose heterogeneity. This study is the first to incorporate JX10 into an NMA framework, filling an evidence gap for this agent in the extended window.

We established a multidimensional evaluation system encompassing functional outcomes, early surrogate endpoints, and safety endpoints, enabling a more comprehensive presentation of benefit-risk profiles. This approach not only confirmed rt-PA's advantages in reperfusion and functional outcomes but also quantified its bleeding risk, providing a refined basis for clinical decision-making. Nevertheless, due to limitations in the number of studies and network structure, findings require validation in larger, rigorously designed head-to-head trials.

### 4.5 Advantages and limitations

Strengths of this study include methodological rigor and forward-looking design: the protocol was prospectively registered (PROSPERO), and reporting followed PRISMA-NMA guidelines. Use of ROB 2.0 and CINeMA for bias assessment and certainty grading ensures reliability and interpretability. Statistical approaches—consistency tests, sensitivity analyses, meta-regression, and multi-indicator ranking—enhance robustness.

However, several limitations should be noted. The evidence for JX10 is extremely weak, based solely on a single small-sample, dose-exploratory study [Niizuma et al., (17); *n* = 90], with no direct comparisons to other thrombolytics. This creates a stark disparity in evidence volume and severely limits robustness. Most trials lacked blinding due to intervention nature, with nine studies assessed as having “some concerns” for bias. Direct evidence for key comparisons (e.g., JX10 vs. TNK) is absent, relying on indirect comparisons, which significantly lowers certainty. Although meta-regression did not identify effect modification, heterogeneity in allowance of concomitant endovascular therapy may affect generalizability. For some outcomes (e.g., 24-h NIHSS, 24-h reperfusion), the number of studies was limited and network structure sparse, precluding thorough inconsistency testing. Given that several outcome measures were informed by fewer than 10 included studies, these analyses should be considered exploratory. This limitation primarily reflects the current scarcity of randomized controlled trials specifically investigating intravenous thrombolysis for acute ischemic stroke in the extended 4.5–24 h time window, where reporting of certain outcomes remains limited. The observation period was limited to 90 days, leaving gaps in evidence on long-term functional maintenance and quality of life-particularly critical in the extended-window population, where baseline severity and recovery trajectories are more complex.

### 4.6 Outlook

As highlighted in a recent Bayesian meta-analysis and umbrella review, the overall evidence base for extended-window thrombolysis remains insufficient to definitively guide clinical decision-making, underscoring the urgent need for further investigation (14). Given current evidence gaps, future research should prioritize: high-quality head-to-head RCTs, particularly direct comparisons between JX10 and rt-PA or TNK, to clarify dose-response and benefit-risk balance; development of standardized core outcome sets for thrombolytic studies, encompassing functional, imaging, safety, and quality-of-life dimensions; extended follow-up periods to capture long-term functional independence and quality of life; real-world studies to explore personalized thrombolytic strategies in subgroups defined by stroke etiology, imaging features, collateral status, and comorbidities; and strengthened trial registration and data sharing to enhance transparency and reproducibility.

## 5 Conclusion

In summary, based on evidence of low to moderate quality, rt-PA remains the most well-established intravenous thrombolytic agent for patients with acute IS within the extended time window of 4.5–24 h. Data indicate significant benefits in terms of reperfusion within 24 h and a non-significant trend toward improved functional independence at 90 days. However, these benefits are offset by a significant increase in the risk of symptomatic intracranial hemorrhage.

In contrast, although a single, small-sample, dose-exploratory trial suggests a potential signal of efficacy for the novel drug JX10, this finding is derived solely from a single-center trial with a small sample size and exploratory dosing, and lacks direct comparative data against standard thrombolytic agents. Therefore, the current evidence regarding JX10 is considered hypothesis-generating only. Its clinical efficacy and safety profile in this setting have not yet been established, and current robust evidence does not support its clinical use.

Tenecteplase demonstrated relatively good safety but failed to demonstrate a clear efficacy advantage over standard therapy in this analysis. Given the low quality of evidence and the urgent need to improve outcomes, clinical decision-making within the extended time window should be individualized and guided by multimodal imaging screening, bleeding risk assessment, and patient preference. There is an urgent need for high-quality, head-to-head RCTs to evaluate the efficacy of JX10 and other novel agents relative to standard therapy.

## Data Availability

The original contributions presented in the study are included in the article/Supplementary material, further inquiries can be directed to the corresponding authors.
