# Supplementary material for: Intravenous thrombolysis for acute ischemic stroke in the extended time window of 4.5–24 h: a systematic review and network meta-analysis of randomized controlled trials
Source: Front Neurol. 2026 May 29;17:1845353. doi: 10.3389/fneur.2026.1845353 (PMC13260050; doi:10.3389/fneur.2026.1845353)
Supplement: Supplementary file 1 [file Table_1.DOCX]

**Intravenous Thrombolysis for Acute Ischemic Stroke in the Extended Time Window of 4.5 to 24 Hours: A Systematic Review and Network Meta-Analysis of Randomized Controlled Trials**

| **Table of Contents** | | |
| --- | --- | --- |
| Title | Content | page |
| Table S1 | PRISMA NMA Checklist of Items to Include When Reporting a Systematic Review Involving a Network Meta-analysis | 2-5 |
| Table S2 | Literature Search Strategy | 6-7 |
| Table S3 | Global inconsistency table for 90d mRS0-2, 36h Sich and 90d Death outcomes in adults with IS | 7 |
| Table S4 | Node-splitting analysis for the 90d mRS0-2 outcome in adults with IS | 7 |
| Table S5 | Node-splitting analysis for the 36h Sich outcome in adults with IS | 8 |
| Table S6 | Node-splitting analysis for the 90d Death outcome in adults with IS | 8 |
| Table S7 | Node-splitting analysis for the 90d Death outcome in adults with IS | 8 |
| Figure S1 | Loop inconsistency plot for the 90d mRS0-2 outcome in adults with IS | 9 |
| Figure S2 | Loop inconsistency plot for the 36h Sich outcome in adults with IS | 9 |
| Figure S3 | Loop inconsistency plot for the 90d Death outcome in adults with IS | 10 |
| Table S8 | SUCRA ranking table for the 90d mRS0-1 outcome in adults with IS | 10 |
| Table S9 | SUCRA ranking table for the 90d mRS0-2 outcome in adults with IS | 10 |
| Table S10 | SUCRA ranking table for the 24h NIHSS outcome in adults with IS | 10 |
| Table S11 | SUCRA ranking table for the 24h Rep outcome in adults with IS | 11 |
| Table S12 | SUCRA ranking table for the 36h Sich outcome in adults with IS | 11 |
| Table S13 | SUCRA ranking table for the 90d Death outcome in adults with IS | 11 |
| Table S14 | Meta-regression analysis of the 90d mRS0-1 outcome with the stroke classification by circulatory territory as a moderator in adults with IS | 11 |
| Table S15 | Meta-regression analysis of the 90d mRS0-1 outcome with the type of EVT as a moderator in adults with IS | 12 |
| Table S16 | Meta-regression analysis of the 90d mRS0-2 outcome with the stroke classification by circulatory territory as a moderator in adults with IS | 12 |
| Table S17 | Meta-regression analysis of the 90d mRS0-2 outcome with the type of EVT as a moderator in adults with IS | 12 |
| Table S18 | Meta-regression analysis of the 24h NIHSS outcome with the stroke classification by circulatory territory as a moderator in adults with IS | 13 |
| Table S19 | Meta-regression analysis of the 24h NIHSS outcome with the type of EVT as a moderator in adults with IS | 13 |
| Table S20 | Meta-regression analysis of the 24h Rep outcome with the stroke classification by circulatory territory as a moderator in adults with IS | 13 |
| Table S21 | Meta-regression analysis of the 24h Rep outcome with the type of EVT as a moderator in adults with IS | 13 |
| Table S22 | Meta-regression analysis of the 36h Sich outcome with the stroke classification by circulatory territory as a moderator in adults with IS | 14 |
| Table S23 | Meta-regression analysis of the 36h Sich outcome with the type of EVT as a moderator in adults with IS | 14 |
| Table S24 | Meta-regression analysis of the 90d Death outcome with the stroke classification by circulatory territory as a moderator in adults with IS | 14 |
| Table S25 | Meta-regression analysis of the 90d Death outcome with the type of EVT as a moderator in adults with IS | 14 |
| Table S26 | Sensitivity analysis for the 90d mRS0-1 outcome in adults with IS | 15 |
| Table S27 | Sensitivity analysis for the 90d mRS0-2 outcome in adults with IS | 16 |
| Table S28 | Sensitivity analysis for the 36h Sich outcome in adults with IS | 17 |
| Table S29 | Sensitivity analysis for the 90d Death outcome in adults with IS | 17 |
| Figure S4 | Comparison-adjusted funnel plot for the 90d mRS0-1 outcome in adults with IS | 18 |
| Figure S5 | Comparison-adjusted funnel plot for the 90d mRS0-2 outcome in adults with IS | 18 |
| Figure S6 | Comparison-adjusted funnel plot for the 24h NIHSS outcome in adults with IS | 19 |
| Figure S7 | Comparison-adjusted funnel plot for the 24h REP outcome in adults with IS | 19 |
| Figure S8 | Comparison-adjusted funnel plot for the 36h Sich outcome in adults with IS | 20 |
| Figure S9 | Comparison-adjusted funnel plot for the 90d DEATH outcome in adults with IS | 20 |
| Table S30 | GRADE certainty of evidence for the 90d mRS0-1 outcome in adults with IS | 21 |
| Table S31 | GRADE certainty of evidence for the 90d mRS0-2 outcome in adults with IS | 22 |
| Table S32 | GRADE certainty of evidence for the 24h NISS outcome in adults with IS | 23 |
| Table S33 | GRADE certainty of evidence for the 24h Sich outcome in adults with IS | 23 |
| Table S34 | GRADE certainty of evidence for the 36h Rep outcome in adults with IS | 24 |
| Table S35 | GRADE certainty of evidence for the 90d Death outcome in adults with IS | 24 |

Table S1 PRISMA NMA Checklist of Items to Include When Reporting a Systematic Review Involving a Network Meta-analysis

| **Section/Topic** | **Item #** | **Checklist Item** | **Reported on Page #** |
| --- | --- | --- | --- |
| TITLE |  |  |  |
| Title | 1 | Identify the report as a systematic review incorporating  anetwork meta-analysis (or related form of meta-analysis). | 1 |
|  |  |  |  |
| ABSTRACT |  |  |  |
| Structured summary | 2 | Provide a structured summary including, as applicable:  Background: main objectives  Methods: data sources; study eligibility criteria, participants, and interventions; study appraisal; and synthesis methods, such as network meta-analysis.  Results: number of studies and participants identified; summary estimates with corresponding confidence/credible intervals; treatment rankings may also be discussed. Authors may choose to summarize pairwise comparisons against a chosen treatment included in their analyses for brevity.  Discussion/Conclusions: limitations; conclusions and implications of findings.  Other: systematic review registration number with registry name. | 1-3 |
|  |  |  |  |
| INTRODUCTION |  |  |  |
| Rationale | 3 | Describe the rationale for the review in the context of what is already known, including mention of why a network meta-analysis has been conducted. | 3-4 |
| Objectives | 4 | Provide an explicit statement of questions being addressed, with reference to participants, interventions, comparisons, outcomes, and study design (PICOS). | 4-5 |
|  |  |  |  |
| METHODS |  |  |  |
| Protocol and registration | 5 | Indicate whether a review protocol exists and if and where it can be accessed (e.g., Web address); and, if available, provide registration information, including registration number. | 6 |
| Eligibility criteria | 6 | Specify study characteristics (e.g., PICOS, length of follow-up) and report characteristics (e.g., years considered, language, publication status) used as criteria for eligibility, giving rationale. Clearly describe eligible treatments included in the treatment network, and note whether any have been clustered or merged into the same node (with justification). | 6 |
| Information sources | 7 | Describe all information sources (e.g., databases with dates of coverage, contact with study authors to identify additional studies) in the search and date last searched. | 6 |
| Search | 8 | Present full electronic search strategy for at least one database, including any limits used, such that it could be repeated. | 6, Supplementary  TableS2 |
| Study selection | 9 | State the process for selecting studies (i.e., screening, eligibility, included in systematic review, and, if applicable, included in the meta-analysis). | 6-7, Table 1 |
| Data collection process | 10 | Describe method of data extraction from reports (e.g., piloted forms, independently, in duplicate) and any processes for obtaining and confirming data from investigators. | 7 |
| Data items | 11 | List and define all variables for which data were sought (e.g., PICOS, funding sources) and any assumptions and simplifications made. | 7 |
| Geometry of the network | S1 | Describe methods used to explore the geometry of the treatment network under study and potential biases related to it. This should include how the evidence base has been graphically summarized for presentation, and what characteristics were compiled and used to describe the evidence base to readers. | 7 |
| Risk of bias within individual studies | 12 | Describe methods used for assessing risk of bias of individual studies (including specification of whether this was done at the study or outcome level), and how this information is to be used in any data synthesis. | 7 |
| Summary measures | 13 | State the principal summary measures (e.g., risk ratio, difference in means). Also describe the use of additional summary measures assessed, such as treatment rankings and surface under the cumulative ranking curve (SUCRA) values, as well as modified approaches used to present summary findings from meta-analyses. | 7 |
| Planned methods of analysis | 14 | Describe the methods of handling data and combining results of studies for each network meta-analysis. This should include, but not be limited to:   - Handling of multi-arm trials; - Selection of variance structure; - Selection of prior distributions in Bayesian analyses; and - Assessment of model fit. | 8-10 |
| Assessment of Inconsistency | S2 | Describe the statistical methods used to evaluate the agreement of direct and indirect evidence in the treatment network(s) studied. Describe efforts taken to address its presence when found. | 10 |
| Risk of bias across studies | 15 | Specify any assessment of risk of bias that may affect the cumulative evidence (e.g., publication bias, selective reporting within studies). | 10 |
| Additional analyses | 16 | Describe methods of additional analyses if done, indicating which were pre-specified. This may include, but not be limited to, the following:   - Sensitivity or subgroup analyses; - Meta-regression analyses; - Alternative formulations of the treatment network; and - Use of alternative prior distributions for Bayesian analyses (if applicable). | 10-11 |
| RESULTS† |  |  |  |
| Study selection | 17 | Give numbers of studies screened, assessed for eligibility, and included in the review, with reasons for exclusions at each stage, ideally with a flow diagram. | 11-12，Figure1 |
| Presentation of network structure | S3 | Provide a network graph of the included studies to enable visualization of the geometry of the treatment network. | 10，Figure3 |
| Summary of network geometry | S4 | Provide a brief overview of characteristics of the treatment network. This may include commentary on the abundance of trials and randomized patients for the different interventions and pairwise comparisons in the network, gaps of evidence in the treatment network, and potential biases reflected by the network structure. | 10 |
| Study characteristics | 18 | For each study, present characteristics for which data were extracted (e.g., study size, PICOS, follow-up period) and provide the citations. | 11-12, Table 1 |
| Risk of bias within studies | 19 | Present data on risk of bias of each study and, if available, any outcome level assessment. | 14, Figure 2 |
| Results of individual studies | 20 | For all outcomes considered (benefits or harms), present, for each study: 1) simple summary data for each intervention group, and 2) effect estimates and confidence intervals. Modified approaches may be needed to deal with information from larger networks. | 16-21 |
| Synthesis of results | 21 | Present results of each meta-analysis done, including confidence/credible intervals. In larger networks, authors may focus on comparisons versus a particular comparator (e.g. placebo or standard care), with full findings presented in an appendix. League tables and forest plots may be considered to summarize pairwise comparisons. If additional summary measures were explored (such as treatment rankings), these should also be presented. | 16-21 |
| Exploration for inconsistency | S5 | Describe results from investigations of inconsistency. This may include such information as measures of model fit to compare consistency and inconsistency models, P values from statistical tests, or summary of inconsistency estimates from different parts of the treatment network. | 17 |
| Risk of bias across studies | 22 | Present results of any assessment of risk of bias across studies for the evidence base being studied. | 17, Supplementary Figure S4-9 |
| Results of additional analyses | 23 | Give results of additional analyses, if done (e.g., sensitivity or subgroup analyses, meta-regression analyses, alternative network geometries studied, alternative choice of prior distributions for Bayesian analyses, and so forth). | 22-23 |
|  |  |  |  |
| DISCUSSION |  |  |  |
| Summary of evidence | 24 | Summarize the main findings, including the strength of evidence for each main outcome; consider their relevance to key groups (e.g., healthcare providers, users, and policy-makers). | 24-28 |
| Limitations | 25 | Discuss limitations at study and outcome level (e.g., risk of bias), and at review level (e.g., incomplete retrieval of identified research, reporting bias). Comment on the validity of the assumptions, such as transitivity and consistency. Comment on any concerns regarding network geometry (e.g., avoidance of certain comparisons). | 28-29 |
| Conclusions | 26 | Provide a general interpretation of the results in the context of other evidence, and implications for future research. | 30 |
|  |  |  |  |
| FUNDING |  |  |  |
| Funding | 27 | Describe sources of funding for the systematic review and other support (e.g., supply of data); role of funders for the systematic review. This should also include information regarding whether funding has been received from manufacturers of treatments in the network and/or whether some of the authors are content experts with professional conflicts of interest that could affect use of treatments in the network. | 30 |

PICOS = population, intervention, comparators, outcomes, study design.

* Text in italics indicate S wording specific to reporting of network meta-analyses that has been added to guidance from the PRISMA statement.

† Authors may wish to plan for use of appendices to present all relevant information in full detail for items in this section.

| **Supplementary Table 2. Literature Search Strategy** | |
| --- | --- |
| Pubmed | \| ID Search Hits  #1 "Ischemic Stroke"[Mesh] 19721  #2 (((((((((((((((((((((((Ischemic Strokes[Title/Abstract]) OR (Stroke, Ischemic[Title/Abstract])) OR (Ischaemic Stroke[Title/Abstract])) OR (Ischaemic Strokes[Title/Abstract])) OR (Stroke, Ischaemic[Title/Abstract])) OR (Acute Ischemic Stroke[Title/Abstract])) OR (Acute Ischemic Strokes[Title/Abstract])) OR (Ischemic Stroke, Acute[Title/Abstract])) OR (Stroke, Acute Ischemic[Title/Abstract])) OR (Cryptogenic Ischemic Stroke[Title/Abstract])) OR (Cryptogenic Ischemic Strokes[Title/Abstract])) OR (Ischemic Stroke, Cryptogenic[Title/Abstract])) OR (Stroke, Cryptogenic Ischemic[Title/Abstract])) OR (Cryptogenic Embolism Stroke[Title/Abstract])) OR (Cryptogenic Embolism Strokes[Title/Abstract])) OR (Embolism Stroke, Cryptogenic[Title/Abstract])) OR (Stroke, Cryptogenic Embolism[Title/Abstract])) OR (Cryptogenic Stroke[Title/Abstract])) OR (Cryptogenic Strokes[Title/Abstract])) OR (Stroke, Cryptogenic[Title/Abstract])) OR (Wake-up Stroke[Title/Abstract])) OR (Stroke, Wake-up[Title/Abstract])) OR (Wake up Stroke[Title/Abstract])) OR (Wake-up Strokes[Title/Abstract]) 45412  #3 #1or#2 54832  #4 "Tissue Plasminogen Activator"[Mesh] 21522  #5 ((((((((((((((((((((Plasminogen Activator, Tissue[Title/Abstract]) OR (Tissue Activator D-44[Title/Abstract])) OR (Tissue Activator D 44[Title/Abstract])) OR (Plasminogen[Title/Abstract])) OR (Activator, Tissue-Type[Title/Abstract])) OR (Plasminogen Activator, Tissue Type[Title/Abstract])) OR (Tissue-Type Plasminogen[Title/Abstract])) OR (Activator[Title/Abstract])) OR (Tissue Type Plasminogen Activator[Title/Abstract])) OR (TTPA[Title/Abstract])) OR (T-Plasminogen Activator[Title/Abstract])) OR (T Plasminogen[Title/Abstract])) OR (Activator[Title/Abstract])) OR (Alteplase[Title/Abstract])) OR (Tisokinase[Title/Abstract])) OR (Actilyse[Title/Abstract])) OR (Lysatec rt-PA[Title/Abstract])) OR (Lysatec rtPA[Title/Abstract])) OR (Lysatec rt PA[Title/Abstract])) OR (Activase[Title/Abstract])) OR (Tenecteplase[Title/Abstract]) 174540  #6 #4or#5 179406  #7 #3and#6 6647  #8 " Randomized Controlled Trial"[Filters] 430 \| \| --- \| |
|  |  |
|  |  |
|  |  |
|  |  |
|  |  |
|  |  |
| Web of Science | ID Search Hits  #1 TS= ("Ischemic Stroke" OR "Ischemic Stroke, Acute" OR "Acute Ischemic Strokes" OR "Acute Ischemic Stroke" OR "Stroke, Acute Ischemic" OR "Stroke, Ischaemic" OR "Ischaemic Stroke" OR "Stroke, Ischemic" OR "Ischaemic Strokes" OR "Ischemic Strokes" OR "Stroke, Wake-up" OR "Wake up Stroke" OR "Wake-up Stroke" OR "Wake-up Strokes" OR "Cryptogenic Ischemic Strokes" OR "Stroke, Cryptogenic Embolism" OR "Ischemic Stroke, Cryptogenic" OR "Cryptogenic Embolism Strokes" OR "Stroke, Cryptogenic" OR "Cryptogenic Ischemic Stroke") 136331  #2 TS= ("Tissue Plasminogen Activator" OR "Tisokinase" OR "T-Plasminogen Activator" OR "T Plasminogen Activator" OR "Tissue Activator D-44" OR "Alteplase" OR "Plasminogen Activator, Tissue-Type" OR "TTPA" OR "Plasminogen Activator, Tissue" OR "Tissue-Type Plasminogen Activator" OR "Tissue Type Plasminogen Activator" OR "Plasminogen Activator, Tissue Type" OR "Tissue Activator D 44" OR "Lysatec rtPA" OR "Actilyse" OR "Lysatec rt PA" OR "Lysatec rt-PA" OR "Activase" OR "Tenecteplase") 38146  #3 TS= ("randomized controlled trial" OR "randomized clinical trial" OR "RCT") 338039  #4 #1 AND #2 AND #3 582 |
|  |  |
|  |  |
|  |  |
|  |  |
|  |  |
|  |  |
|  |  |
| Cochrane | ID Search Hits  #1 MeSH descriptor: [Ischemic Stroke] explode all trees 2186  #2 ("Ischemic Stroke, Acute" OR "Acute Ischemic Strokes" OR "Acute Ischemic Stroke" OR "Stroke, Acute Ischemic" OR "Stroke, Ischaemic" OR "Ischaemic Stroke" OR "Stroke, Ischemic" OR "Ischaemic Strokes" OR "Ischemic Strokes" OR "Stroke, Wake-up" OR "Wake up Stroke" OR "Wake-up Stroke" OR "Wake-up Strokes" OR "Cryptogenic Ischemic Strokes" OR "Stroke, Cryptogenic Embolism" OR "Ischemic Stroke, Cryptogenic" OR "Cryptogenic Embolism Strokes" OR "Stroke, Cryptogenic" OR "Cryptogenic Ischemic Stroke"):ti,ab,kw 16069  #3 #1 OR #2 16134  #4 MeSH descriptor: [Tissue Plasminogen Activator] explode all trees 2492  #5 ("Tisokinase" OR "T-Plasminogen Activator" OR "T Plasminogen Activator" OR "Tissue Activator D-44" OR "Alteplase" OR "Plasminogen Activator, Tissue-Type" OR "TTPA" OR "Plasminogen Activator, Tissue" OR "Tissue-Type Plasminogen Activator" OR "Tissue Type Plasminogen Activator" OR "Plasminogen Activator, Tissue Type" OR "Tissue Activator D 44" OR "Lysatec rtPA" OR "Actilyse" OR "Lysatec rt PA" OR "Lysatec rt-PA" OR "Activase" OR "Tenecteplase"):ti,ab,kw 2684  #6 #4 OR #5 3934  #7 #3 AND #6 1411 |
|  |  |
|  |  |
|  |  |
| Embase | ID Search Hits  #1 'ischemic stroke'/exp OR 'ischemic stroke' OR 'ischaemic stroke':ab,ti,kw OR 'ischemic stroke':ab,ti,kw 167191  #2 'tissue plasminogen activator'/exp OR 'ak 124':ab,ti,kw OR 'ak124':ab,ti,kw OR 'angiochinase':ab,ti,kw OR 'angiokinase':ab,ti,kw OR 'hapase':ab,ti,kw OR 'plasminogen activator, tissue':ab,ti,kw OR 'plasminogen activator, tissue type':ab,ti,kw OR 'plasvata':ab,ti,kw OR 'plasminogen activator':ab,ti,kw OR 'tisokinase':ab,ti,kw OR 'tissue type plasminogen activator':ab,ti,kw OR 'tissue plasminogen activator':ab,ti,kw OR 'alteplase':ab,ti,kw OR 'tenecteplase':ab,ti,kw 77954  #3 #1 AND #2 14230  #4 #3 AND 'randomized controlled trial topic'/de 636 |
|  |  |
|  |  |
|  |  |
|  |  |
|  |  |

Supplementary Table 3. Global inconsistency table for 90d mRS0-2, 36h Sich and 90d Death outcomes in adults with IS

| **Outcome measure name** | **P-value for global inconsistency analysis** |
| --- | --- |
| 90d mRS0-2 | 0.6131 |
| 36h Sich | 0.3414 |
| 90d Death | 0.5450 |

Supplementary Table 4. Node-splitting analysis for the 90d mRS0-2 outcome in adults with IS

|  | **Coef.** | **Std. Err.** | **Coef.** | **Std. Err.** | **Coef.** | **Std. Err.** | **P>z** | **tau** |
| --- | --- | --- | --- | --- | --- | --- | --- | --- |
| A B | -0.3794896 | 0.2562492 | -0.0465273 | 90.36843 | -0.3329622 | 90.36879 | 0.997 | 0.0637758 |
| B D | 0.082867 | 0.1367432 | -0.0309956 | 0.1789037 | 0.1138626 | 0.2251782 | 0.613 | 0.0869807 |
| B E | 0.1205936 | 0.1334834 | 0.2344587 | 0.181219 | -0.113865 | 0.2251797 | 0.613 | 0.0869821 |
| C D | 0.0509675 | 0.083673 | 0.1648313 | 0.2089955 | -0.1138637 | 0.2251797 | 0.613 | 0.086982 |
| C E | 0.2025585 | 0.0847004 | 0.0886939 | 0.2087533 | 0.1138646 | 0.2251805 | 0.613 | 0.0869819 |

Supplementary Table 5. Node-splitting analysis for the 36h Sich outcome in adults with IS

|  | **Coef.** | **Std. Err.** | **Coef.** | **Std. Err.** | **Coef.** | **Std. Err.** | **P>z** | **tau** |
| --- | --- | --- | --- | --- | --- | --- | --- | --- |
| A B | -0.3794896 | 0.2562492 | -0.0465273 | 90.36843 | -0.3329622 | 90.36879 | 0.997 | 0.0637758 |
| B D | 0.082867 | 0.1367432 | -0.0309956 | 0.1789037 | 0.1138626 | 0.2251782 | 0.613 | 0.0869807 |
| B E | 0.1205936 | 0.1334834 | 0.2344587 | 0.181219 | -0.113865 | 0.2251797 | 0.613 | 0.0869821 |
| C D | 0.0509675 | 0.083673 | 0.1648313 | 0.2089955 | -0.1138637 | 0.2251797 | 0.613 | 0.086982 |
| C E | 0.2025585 | 0.0847004 | 0.0886939 | 0.2087533 | 0.1138646 | 0.2251805 | 0.613 | 0.0869819 |

Supplementary Table 6. Node-splitting analysis for the 90d Death outcome in adults with IS

|  | **Coef.** | **Std.Err.** | **Coef.** | **Std.Err.** | **Coef.** | **Std. Err.** | **P>z** | **tau** |
| --- | --- | --- | --- | --- | --- | --- | --- | --- |
| A C | 0.0791194 | 0.1990899 | 0.4182162 | 0.5237004 | -0.3390967 | 0.5602668 | 0.545 | 0.0000101 |
| A D | 0.2534753 | 0.3989298 | -0.0856275 | 0.3933879 | 0.3391028 | 0.5602669 | 0.545 | 0.0000274 |
| B C | 0.0346742 | 0.2222355 | -0.3044259 | 0.5143057 | 0.3391001 | 0.5602669 | 0.545 | 6.13E-08 |
| B D | -0.1300701 | 0.2563761 | 0.2090301 | 0.498167 | -0.3391002 | 0.5602669 | 0.545 | 5.50E-07 |

Supplementary Table 7. Node-splitting analysis for the 90d Death outcome in adults with IS

| **90d mRS0-1** | **τ** | **90d mRS0-2** | **τ** | **36h Sich** | **τ** |
| --- | --- | --- | --- | --- | --- |
| JX10 VS PBO | 0.06183757 | JX10 VS PBO | 0.06376876 | JX10 VS PBO | 0.00001212 |
| JX10 VS SoC | 0.06183757 | JX10 VS SoC | 0.06376876 | JX10 VS SoC | 0.00001212 |
| JX10 VS TNK | 0.06183757 | JX10 VS TNK | 0.06376876 | JX10 VS TNK | 0.00001212 |
| JX10 VS rt-PA | 0.06183757 | JX10 VS rt-PA | 0.06376876 | JX10 VS rt-PA | 0.00001212 |
| 24h NIHSS | τ | 24h Rep | τ | 90d Death | τ |
| PBO VS SoC | 0.26499965 | PBO VS SoC | 0.000001117 | PBO VS SoC | 2.987E-08 |
| PBO VS TNK | 0.26499965 | PBO VS TNK | 0.000001117 | PBO VS TNK | 2.987E-08 |
| PBO VS rt-PA | 0.26499965 | PBO VS rt-PA | 0.000001117 | PBO VS rt-PA | 2.987E-08 |


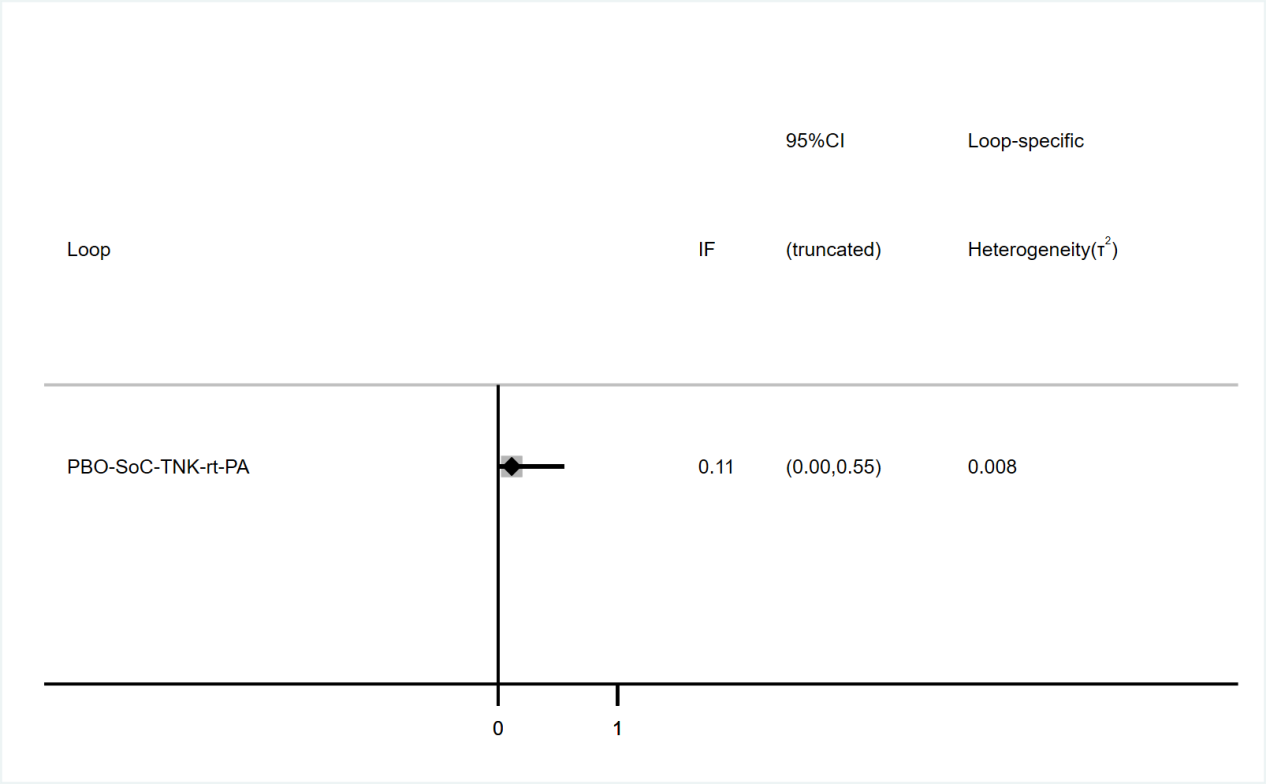


Supplementary Figure1. Loop inconsistency plot for the 90d mRS0-2 outcome in adults with IS
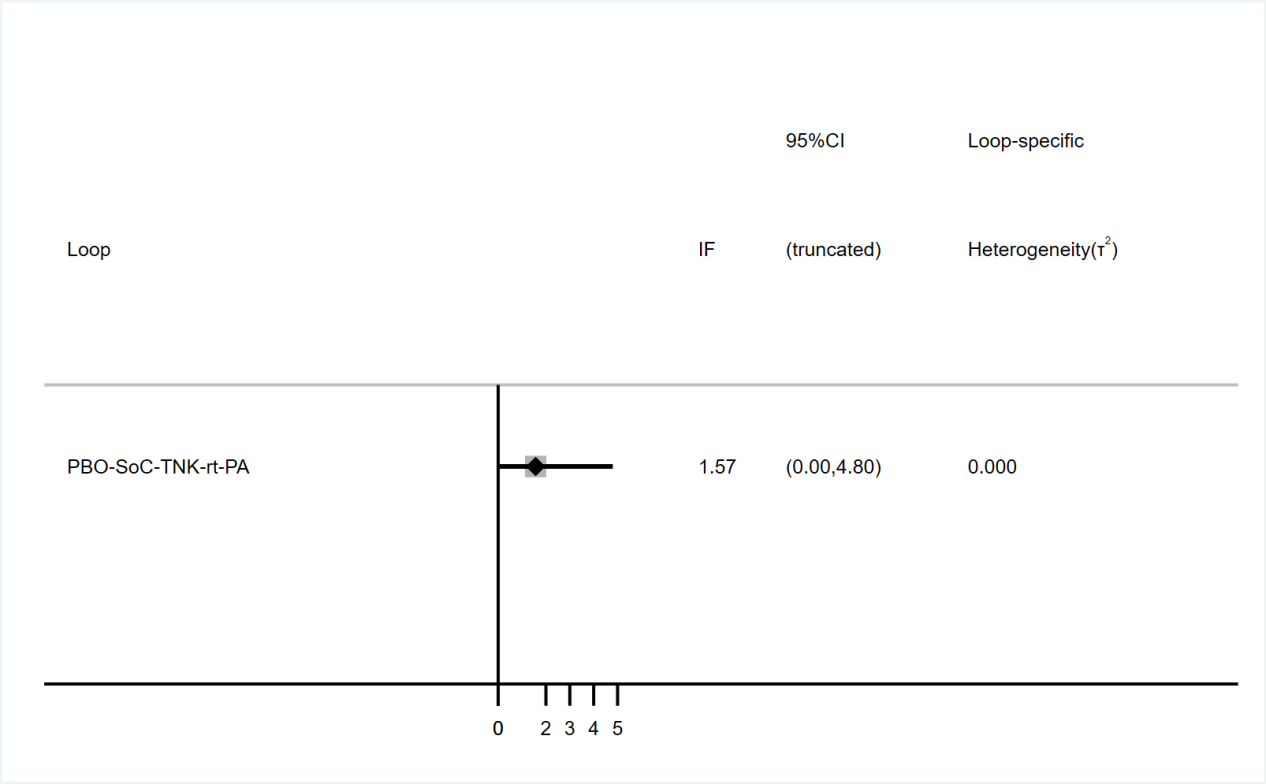
Supplementary Figure 2. Loop inconsistency plot for the 36h Sich outcome in adults with IS


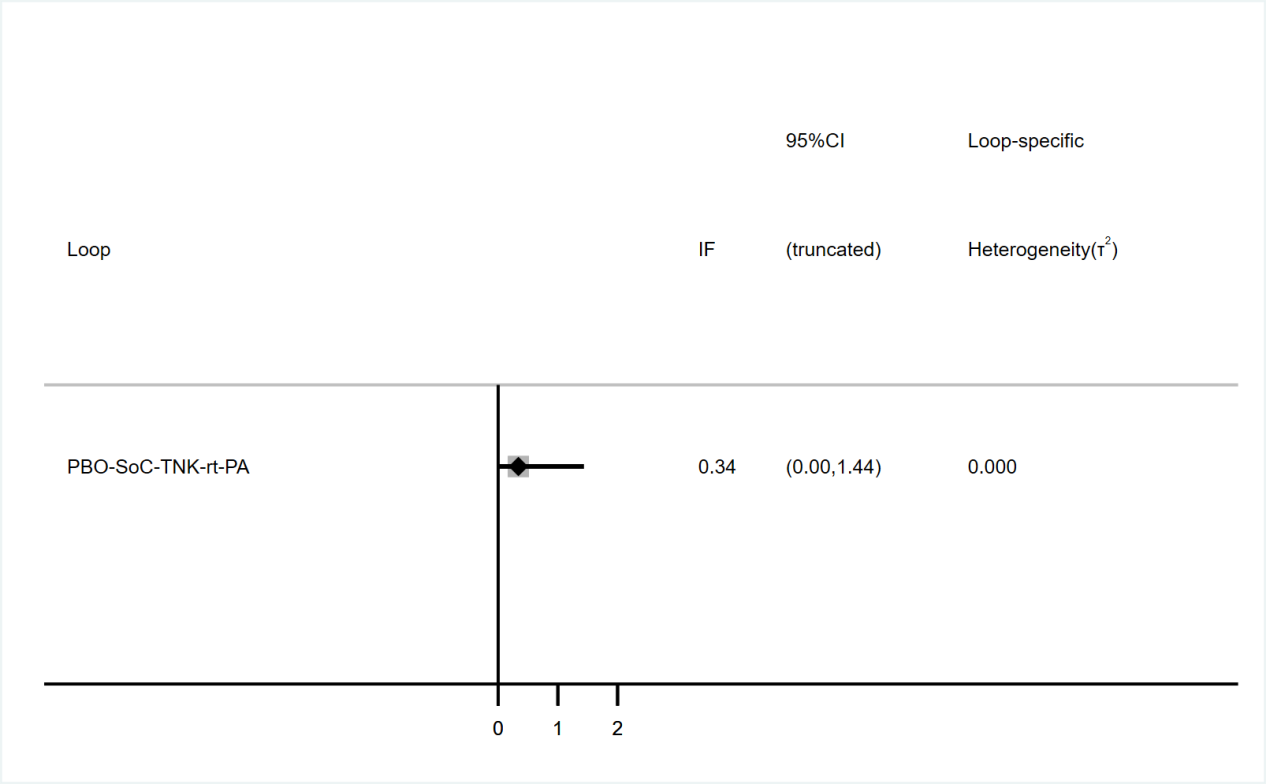


Supplementary Figure 3. Loop inconsistency plot for the 90d Death outcome in adults with IS

Supplementary Table 8. SUCRA ranking table for the 90d mRS0-1 outcome in adults with IS

| **Treatm~t** | **SUCRA** | **PrBest** | **MeanRank** |
| --- | --- | --- | --- |
| JX10 | 95.3 | 90.8 | 1.2 |
| rt-PA | 69.3 | 6.9 | 2.2 |
| TNK | 43.6 | 2.2 | 3.3 |
| PBO | 30.4 | 0.2 | 3.8 |
| SoC | 11.4 | 0 | 4.5 |

Supplementary Table9. SUCRA ranking table for the 90d mRS0-2 outcome in adults with IS

| **Treatm~t** | **SUCRA** | **PrBest** | **MeanRank** |
| --- | --- | --- | --- |
| JX10 | 87.7 | 77.2 | 1.5 |
| rt-PA | 77 | 20.9 | 1.9 |
| TNK | 42.4 | 1.6 | 3.3 |
| PBO | 26.6 | 0.3 | 3.9 |
| SoC | 16.3 | 0 | 4.3 |

Supplementary Table10.SUCRA ranking table for the 24h NIHSS outcome in adults with IS

| **Treatm~t** | **SUCRA** | **PrBest** | **MeanRank** |
| --- | --- | --- | --- |
| rt-PA | 77.2 | 48.2 | 1.7 |
| TNK | 73.1 | 46.5 | 1.8 |
| SoC | 44.3 | 4.3 | 2.7 |
| PBO | 5.5 | 1.1 | 3.8 |

Supplementary Table11. SUCRA ranking table for the 24h Rep outcome in adults with IS

| **Treatm~t** | **SUCRA** | **PrBest** | **MeanRank** |
| --- | --- | --- | --- |
| rt-PA | 99.9 | 99.6 | 1 |
| PBO | 51.2 | 0.1 | 2.5 |
| TNK | 47.8 | 0.2 | 2.6 |
| SoC | 1.1 | 0 | 4 |

Supplementary Table12.SUCRA ranking table for the 36h Sich outcome in adults with IS

| **Treatm~t** | **SUCRA** | **PrBest** | **MeanRank** |
| --- | --- | --- | --- |
| JX10 | 82.1 | 63.2 | 1.7 |
| SoC | 74.7 | 31.1 | 2 |
| PBO | 55.6 | 4.8 | 2.8 |
| TNK | 30.2 | 0.8 | 3.8 |
| rt-PA | 7.4 | 0.1 | 4.7 |

Supplementary Table13.SUCRA ranking table for the 90d Death outcome in adults with IS

| **Treatm~t** | **SUCRA** | **PrBest** | **MeanRank** |
| --- | --- | --- | --- |
| PBO | 69.1 | 46.6 | 1.9 |
| rt-PA | 52.2 | 30 | 2.4 |
| TNK | 40.1 | 10.5 | 2.8 |
| SoC | 38.6 | 13 | 2.8 |

Supplementary Table 14.Meta-regression analysis of the 90d mRS0-1 outcome with the stroke classification by circulatory territory as a moderator in adults with IS

| **Intervention** | **Covariate** | **Coefficient** | **Standard Error** | **Z-statistic** | **P>z** | **lower confidence interval** | **upper confidence interval** |
| --- | --- | --- | --- | --- | --- | --- | --- |
| JX10 VS PBO | stroke classification by circulatory territory | 0.4910477 | 95.99759 | 0.01 | 0.996 | -187.6608 | 188.6429 |
| JX10 VS PBO | Intercept | -1.276001 | 95.99929 | -0.01 | 0.989 | -189.4312 | 186.8792 |
| JX10 VS SoC | stroke classification by circulatory territory | 0.2528631 | 95.9978 | 0 | 0.998 | -187.8994 | 188.4051 |
| JX10 VS SoC | Intercept | -0.8036317 | 96.00174 | -0.01 | 0.993 | -188.9636 | 187.3563 |
| JX10 VS TNK | stroke classification by circulatory territory | 0.2052226 | 95.99781 | 0 | 0.998 | -187.947 | 188.3575 |
| JX10 VS TNK | Intercept | -0.6119199 | 96.00183 | -0.01 | 0.995 | -188.7721 | 187.5482 |
| JX10 VS rt-PA | stroke classification by circulatory territory | 0.4813183 | 95.99767 | 0.01 | 0.996 | -187.6707 | 188.6333 |
| JX10 VS rt-PA | Intercept | -1.063329 | 96.00033 | -0.01 | 0.991 | -189.2205 | 187.0939 |

Supplementary Table 15.Meta-regression analysis of the 90d mRS0-1 outcome with the type of EVT as a moderator in adults with IS

| **Intervention** | **Covariate** | **Coefficient** | **Standard Error** | **Z-statistic** | **P>z** | **lower confidence interval** | **upper confidence interval** |
| --- | --- | --- | --- | --- | --- | --- | --- |
| JX10 VS PBO | Intercept | -0.7849532 | 0.3897568 | -2.01 | 0.044 | -1.548863 | -0.0210439 |
| JX10 VS SoC | type of EVT | -0.2762179 | 176.9178 | 0 | 0.999 | -347.0288 | 346.4763 |
| JX10 VS SoC | Intercept | 0.4094799 | 372.9643 | 0 | 0.999 | -730.5871 | 731.4061 |
| JX10 VS TNK | type of EVT | -0.3416723 | 176.9178 | 0 | 0.998 | -347.0943 | 346.4109 |
| JX10 VS TNK | Intercept | 0.6868789 | 372.9644 | 0 | 0.999 | -730.3099 | 731.6837 |
| JX10 VS rt-PA | type of EVT | 0.7270711 | 144.7881 | 0.01 | 0.996 | -283.0524 | 284.5065 |
| JX10 VS rt-PA | Intercept | -1.321724 | 144.7901 | -0.01 | 0.993 | -285.1051 | 282.4617 |

Supplementary Table 16.Meta-regression analysis of the 90d mRS0-2 outcome with the stroke classification by circulatory territory as a moderator in adults with IS

| **Intervention** | **Covariate** | **Coefficient** | **Standard Error** | **Z-statistic** | **P>z** | **lower confidence interval** | **upper confidence interval** |
| --- | --- | --- | --- | --- | --- | --- | --- |
| JX10 VS PBO | stroke classification by circulatory territory | 0.2123156 | 81.28381 | 0 | 0.998 | -159.101 | 159.5256 |
| JX10 VS PBO | Intercept | -0.5918037 | 81.28519 | -0.01 | 0.994 | -159.9078 | 158.7242 |
| JX10 VS SoC | stroke classification by circulatory territory | 0.1714569 | 81.28388 | 0 | 0.998 | -159.142 | 159.4849 |
| JX10 VS SoC | Intercept | -0.5416649 | 81.28585 | -0.01 | 0.995 | -159.859 | 158.7757 |
| JX10 VS TNK | stroke classification by circulatory territory | 0.1876641 | 81.28388 | 0 | 0.998 | -159.1258 | 159.5012 |
| JX10 VS TNK | Intercept | -0.510244 | 81.28569 | -0.01 | 0.995 | -159.8273 | 158.8068 |
| JX10 VS rt-PA | stroke classification by circulatory territory | 0.2204453 | 81.28386 | 0 | 0.998 | -159.093 | 159.5339 |
| JX10 VS rt-PA | Intercept | -0.4709309 | 81.28589 | -0.01 | 0.995 | -159.7884 | 158.8465 |

Supplementary Table 17.Meta-regression analysis of the 90d mRS0-2 outcome with the type of EVT as a moderator in adults with IS

| **Intervention** | **Covariate** | **Coefficient** | **Standard Error** | **Z-statistic** | **P>z** | **lower confidence interval** | **upper confidence interval** |
| --- | --- | --- | --- | --- | --- | --- | --- |
| JX10 VS PBO | type of EVT | 0.3205881 | 70.74255 | 0 | 0.996 | -138.3323 | 138.9734 |
| JX10 VS PBO | Intercept | -0.700077 | 70.74339 | -0.01 | 0.992 | -139.3546 | 137.9544 |
| JX10 VS SoC | type of EVT | 0.7240908 | 100.633 | 0.01 | 0.994 | -196.5129 | 197.961 |
| JX10 VS SoC | Intercept | -1.77432 | 214.8291 | -0.01 | 0.993 | -422.8317 | 419.2831 |
| JX10 VS TNK | type of EVT | 0.5633823 | 100.6329 | 0.01 | 0.996 | -196.6735 | 197.8003 |
| JX10 VS TNK | Intercept | -1.345591 | 214.8291 | -0.01 | 0.995 | -422.4029 | 419.7117 |
| JX10 VS rt-PA | type of EVT | 0.1351489 | 92.06109 | 0 | 0.999 | -180.3013 | 180.5716 |
| JX10 VS rt-PA | Intercept | -0.3919941 | 92.06221 | 0 | 0.997 | -180.8306 | 180.0466 |

Supplementary Table 18.Meta-regression analysis of the 24h NIHSS outcome with the stroke classification by circulatory territory as a moderator in adults with IS

| **Intervention** | **Covariate** | **Coefficient** | **Standard Error** | **Z-statistic** | **P>z** | **lower confidence interval** | **upper confidence interval** |
| --- | --- | --- | --- | --- | --- | --- | --- |
| PBO VS SoC | stroke classification by circulatory territory | 0.2697569 | 102.1677 | 0 | 0.998 | -199.9753 | 200.5148 |
| PBO VS SoC | Intercept | -0.4057315 | 306.5028 | 0 | 0.999 | -601.1401 | 600.3287 |
| PBO VS TNK | Intercept | 0.0628762 | 204.3352 | 0 | 1 | -400.4267 | 400.5525 |
| PBO VS rt-PA | stroke classification by circulatory territory | 0.7466819 | 102.1678 | 0.01 | 0.994 | -199.4985 | 200.9919 |
| PBO VS rt-PA | Intercept | -1.350995 | 306.5032 | 0 | 0.996 | -602.0861 | 599.3842 |

Supplementary Table 19.Meta-regression analysis of the 24h NIHSS outcome with the type of EVT as a moderator in adults with IS

| **Intervention** | **Covariate** | **Coefficient** | **Standard Error** | **Z-statistic** | **P>z** | **lower confidence interval** | **upper confidence interval** |
| --- | --- | --- | --- | --- | --- | --- | --- |
| PBO VS SoC | type of EVT | 0.0164521 | 488.9418 | 0 | 1 | -958.2918 | 958.3247 |
| PBO VS SoC | Intercept | -0.1504944 | 1302.702 | 0 | 1 | -2553.4 | 2553.099 |
| PBO VS TNK | Intercept | 0.0977127 | 294.9342 | 0 | 1 | -577.9627 | 578.1581 |
| PBO VS rt-PA | type of EVT | -0.7869637 | 381.4979 | 0 | 0.998 | -748.5092 | 746.9352 |
| PBO VS rt-PA | Intercept | 1.676017 | 381.4987 | 0 | 0.996 | -746.0477 | 749.3997 |

Supplementary Table 20.Meta-regression analysis of the 24h Rep outcome with the stroke classification by circulatory territory as a moderator in adults with IS

| **Intervention** | **Covariate** | **Coefficient** | **Standard Error** | **Z-statistic** | **P>z** | **lower confidence interval** | **upper confidence interval** |
| --- | --- | --- | --- | --- | --- | --- | --- |
| PBO VS SoC | Intercept | -0.4102646 | 0.1950232 | -2.1 | 0.035 | -0.7925031 | -0.0280261 |
| PBO VS TNK | Intercept | -0.0138891 | 0.0915597 | -0.15 | 0.879 | -0.1933429 | 0.1655647 |
| PBO VS rt-PA | Intercept | 0.5642135 | 0.1803267 | 3.13 | 0.002 | 0.2107797 | 0.9176473 |

Supplementary Table 21.Meta-regression analysis of the 24h Rep outcome with the type of EVT as a moderator in adults with IS

| **Intervention** | **Covariate** | **Coefficient** | **Standard Error** | **Z-statistic** | **P>z** | **lower confidence interval** | **upper confidence interval** |
| --- | --- | --- | --- | --- | --- | --- | --- |
| PBO VS SoC | type of EVT | 0.1020345 | 88.39828 | 0 | 0.999 | -173.1554 | 173.3595 |
| PBO VS SoC | Intercept | -0.5109804 | 265.1942 | 0 | 0.998 | -520.2821 | 519.2601 |
| PBO VS TNK | type of EVT | -0.2396071 | 88.39773 | 0 | 0.998 | -173.496 | 173.0168 |
| PBO VS TNK | Intercept | 0.704928 | 265.1932 | 0 | 0.998 | -519.0642 | 520.474 |
| PBO VS rt-PA | Intercept | 0.5642126 | 0.1844965 | 3.06 | 0.002 | 0.2026061 | 0.9258191 |

Supplementary Table 22.Meta-regression analysis of the 36h Sich outcome with the stroke classification by circulatory territory as a moderator in adults with IS

| **Intervention** | **Covariate** | **Coefficient** | **Standard Error** | **Z-statistic** | **P>z** | **lower confidence interval** | **upper confidence interval** |
| --- | --- | --- | --- | --- | --- | --- | --- |
| JX10 VS PBO | stroke classification by circulatory territory | -1.189545 | 637.4163 | 0 | 0.999 | -1250.503 | 1248.123 |
| JX10 VS PBO | Intercept | 2.215391 | 637.4188 | 0 | 0.997 | -1247.102 | 1251.533 |
| JX10 VS SoC | stroke classification by circulatory territory | -0.6749044 | 637.4165 | 0 | 0.999 | -1249.988 | 1248.638 |
| JX10 VS SoC | Intercept | 0.6789278 | 637.4204 | 0 | 0.999 | -1248.642 | 1250 |
| JX10 VS TNK | Intercept | 1.343798 | 1.337327 | 1 | 0.315 | -1.277314 | 3.964911 |
| JX10 VS rt-PA | stroke classification by circulatory territory | 0.5701675 | 637.4177 | 0 | 0.999 | -1248.746 | 1249.886 |
| JX10 VS rt-PA | Intercept | -1.126726 | 637.4327 | 0 | 0.999 | -1250.472 | 1248.218 |

Supplementary Table 23.Meta-regression analysis of the 36h Sich outcome with the type of EVT as a moderator in adults with IS

| **Intervention** | **Covariate** | **Coefficient** | **Standard Error** | **Z-statistic** | **P>z** | **lower confidence interval** | **upper confidence interval** |
| --- | --- | --- | --- | --- | --- | --- | --- |
| JX10 VS PBO | type of EVT | -0.6120109 | 747.1825 | 0 | 0.999 | -1465.063 | 1463.839 |
| JX10 VS PBO | Intercept | 1.637863 | 747.1846 | 0 | 0.998 | -1462.817 | 1466.093 |
| JX10 VS SoC | Intercept | -0.9271804 | 1141.5 | 0 | 0.999 | -2238.225 | 2236.371 |
| JX10 VS TNK | type of EVT | -0.2928108 | 1209.251 | 0 | 1 | -2370.382 | 2369.796 |
| JX10 VS TNK | Intercept | 0.9982155 | 2866.452 | 0 | 1 | -5617.144 | 5619.141 |
| JX10 VS rt-PA | type of EVT | -2.499521 | 1141.501 | 0 | 0.998 | -2239.8 | 2234.801 |
| JX10 VS rt-PA | Intercept | 5.462393 | 1141.504 | 0 | 0.996 | -2231.844 | 2242.769 |

Supplementary Table 24.Meta-regression analysis of the 90d Death outcome with the stroke classification by circulatory territory as a moderator in adults with IS

| **Intervention** | **Covariate** | **Coefficient** | **Standard Error** | **Z-statistic** | **P>z** | **lower confidence interval** | **upper confidence interval** |
| --- | --- | --- | --- | --- | --- | --- | --- |
| PBO VS SoC | stroke classification by circulatory territory | 0.104515 | 0.2904383 | 0.36 | 0.719 | -0.4647336 | 0.6737636 |
| PBO VS SoC | Intercept | -0.0600698 | 0.512255 | -0.12 | 0.907 | -1.064071 | 0.9439316 |
| PBO VS TNK | Intercept | 0.0791194 | 0.1990899 | 0.4 | 0.691 | -0.3110896 | 0.4693284 |
| PBO VS rt-PA | stroke classification by circulatory territory | 0.5980988 | 0.5777355 | 1.04 | 0.301 | -0.534242 | 1.73044 |
| PBO VS rt-PA | Intercept | -1.540821 | 1.638815 | -0.94 | 0.347 | -4.75284 | 1.671197 |

Supplementary Table 25.Meta-regression analysis of the 90d Death outcome with the type of EVT as a moderator in adults with IS

| **Intervention** | **Covariate** | **Coefficient** | **Standard Error** | **Z-statistic** | **P>z** | **lower confidence interval** | **upper confidence interval** |
| --- | --- | --- | --- | --- | --- | --- | --- |
| PBO VS SoC | type of EVT | -0.6468256 | 393.7656 | 0 | 0.999 | -772.4133 | 771.1196 |
| PBO VS SoC | Intercept | 1.346298 | 1181.294 | 0 | 0.999 | -2313.947 | 2316.639 |
| PBO VS TNK | type of EVT | 0.0141563 | 393.7638 | 0 | 1 | -771.7486 | 771.777 |
| PBO VS TNK | Intercept | 0.0366382 | 1181.291 | 0 | 1 | -2315.252 | 2315.325 |
| PBO VS rt-PA | type of EVT | -0.330929 | 393.764 | 0 | 0.999 | -772.0942 | 771.4324 |

Supplementary Table 26.Sensitivity analysis for the 90d mRS0-1 outcome in adults with IS

| **dropped_id** | **comparison** | **RR** | **RR lci** | **RR uci** | **connected** |
| --- | --- | --- | --- | --- | --- |
| Ma 2019 | SoC VS JX10 | 2.3571038 | 0.8915557 | 6.231734 | 1 |
| Ma 2019 | SoC VS PBO | 1.0751705 | 0.5893123 | 1.961594 | 1 |
| Ma 2019 | SoC VS TNK | 1.1412846 | 0.9234295 | 1.410536 | 1 |
| Ma 2019 | SoC VS rt-PA | 1.3170842 | 1.067324 | 1.625291 | 1 |
| Zhou 2025 | SoC VS JX10 | 2.2083153 | 0.9645268 | 5.056009 | 1 |
| Zhou 2025 | SoC VS PBO | 1.007302 | 0.7029676 | 1.443391 | 1 |
| Zhou 2025 | SoC VS TNK | 1.1552722 | 0.9638602 | 1.384697 | 1 |
| Zhou 2025 | SoC VS rt-PA | 1.2180036 | 1.0155 | 1.460889 | 1 |
| Yan 2025 | SoC VS JX10 | 2.7750929 | 1.173079 | 6.564893 | 1 |
| Yan 2025 | SoC VS PBO | 1.2658324 | 0.8236758 | 1.945343 | 1 |
| Yan 2025 | SoC VS TNK | 1.1552722 | 0.9638602 | 1.384697 | 1 |
| Yan 2025 | SoC VS rt-PA | 1.530612 | 1.137112 | 2.060284 | 1 |
| Yogendrakumar 2025 | SoC VS JX10 | 2.3606333 | 1.024663 | 5.43846 | 1 |
| Yogendrakumar 2025 | SoC VS PBO | 1.0767805 | 0.7467945 | 1.552577 | 1 |
| Yogendrakumar 2025 | SoC VS TNK | 1.2007546 | 0.9830641 | 1.466651 | 1 |
| Yogendrakumar 2025 | SoC VS rt-PA | 1.3021266 | 1.081039 | 1.56843 | 1 |
| Xiong 2024 | SoC VS JX10 | 2.350106 | 1.032071 | 5.351376 | 1 |
| Xiong 2024 | SoC VS PBO | 1.0719785 | 0.7576987 | 1.516616 | 1 |
| Xiong 2024 | SoC VS TNK | 1.0236133 | 0.8062181 | 1.299629 | 1 |
| Xiong 2024 | SoC VS rt-PA | 1.2962089 | 1.10998 | 1.513683 | 1 |
| Cheng 2025 | SoC VS JX10 | 2.3919068 | 1.006095 | 5.686558 | 1 |
| Cheng 2025 | SoC VS PBO | 1.0910456 | 0.730492 | 1.62956 | 1 |
| Cheng 2025 | SoC VS TNK | 1.1589522 | 0.8910849 | 1.507343 | 1 |
| Cheng 2025 | SoC VS rt-PA | 1.3198231 | 1.062171 | 1.639974 | 1 |
| Niizuma 2024 | SoC VS PBO | 1.0826729 | 0.7424118 | 1.578882 | 1 |
| Niizuma 2024 | SoC VS TNK | 1.1469787 | 0.9373454 | 1.403496 | 1 |
| Niizuma 2024 | SoC VS rt-PA | 1.3094116 | 1.077105 | 1.591822 | 1 |
| Ringleb 2019 | SoC VS JX10 | 2.4034176 | 0.9831916 | 5.875168 | 1 |
| Ringleb 2019 | SoC VS PBO | 1.0962961 | 0.6893257 | 1.743537 | 1 |
| Ringleb 2019 | SoC VS TNK | 1.1412844 | 0.923446 | 1.41051 | 1 |
| Ringleb 2019 | SoC VS rt-PA | 1.3170843 | 1.067347 | 1.625255 | 1 |
| Wang 2023 | SoC VS JX10 | 2.3839538 | 1.012846 | 5.611156 | 1 |
| Wang 2023 | SoC VS PBO | 1.0874179 | 0.7361258 | 1.606353 | 1 |
| Wang 2023 | SoC VS TNK | 1.1660665 | 0.923427 | 1.472462 | 1 |
| Wang 2023 | SoC VS rt-PA | 1.3153019 | 1.068718 | 1.61878 | 1 |

Supplementary Table 27.Sensitivity analysis for the 90d mRS0-2 outcome in adults with IS

| **dropped_id** | **comparison** | **RR** | **RR lci** | **RR uci** | **connected** |
| --- | --- | --- | --- | --- | --- |
| Ma 2019 | SoC VS JX10 | 1.4873433 | 0.8361871 | 2.645568 | 1 |
| Ma 2019 | SoC VS PBO | 1.017656 | 0.7861808 | 1.317285 | 1 |
| Ma 2019 | SoC VS TNK | 1.066524 | 0.9124107 | 1.246668 | 1 |
| Ma 2019 | SoC VS rt-PA | 1.2078388 | 1.030491 | 1.415708 | 1 |
| Zhou 2025 | SoC VS JX10 | 1.4960786 | 0.8381043 | 2.670612 | 1 |
| Zhou 2025 | SoC VS PBO | 1.0236328 | 0.8014307 | 1.307442 | 1 |
| Zhou 2025 | SoC VS TNK | 1.0665397 | 0.9075286 | 1.253412 | 1 |
| Zhou 2025 | SoC VS rt-PA | 1.1984483 | 0.9729291 | 1.476241 | 1 |
| Yan 2025 | SoC VS JX10 | 1.4774056 | 0.8238934 | 2.649283 | 1 |
| Yan 2025 | SoC VS PBO | 1.0108565 | 0.7835603 | 1.304087 | 1 |
| Yan 2025 | SoC VS TNK | 1.0629806 | 0.9035851 | 1.250494 | 1 |
| Yan 2025 | SoC VS rt-PA | 1.1736477 | 0.9254051 | 1.488482 | 1 |
| Yogendrakumar 2025 | SoC VS JX10 | 1.518226 | 0.8782692 | 2.624491 | 1 |
| Yogendrakumar 2025 | SoC VS PBO | 1.0387863 | 0.8424264 | 1.280915 | 1 |
| Yogendrakumar 2025 | SoC VS TNK | 1.0933659 | 0.9361941 | 1.276924 | 1 |
| Yogendrakumar 2025 | SoC VS rt-PA | 1.2111234 | 1.052306 | 1.39391 | 1 |
| Xiong 2024 | SoC VS JX10 | 1.4219127 | 0.8451247 | 2.392352 | 1 |
| Xiong 2024 | SoC VS PBO | 0.97288772 | 0.8089392 | 1.170064 | 1 |
| Xiong 2024 | SoC VS TNK | 0.98294129 | 0.8442157 | 1.144463 | 1 |
| Xiong 2024 | SoC VS rt-PA | 1.2028117 | 1.084819 | 1.333638 | 1 |
| Albers 2024 | SoC VS JX10 | 1.5863792 | 0.8694233 | 2.894561 | 1 |
| Albers 2024 | SoC VS PBO | 1.0854175 | 0.796271 | 1.479561 | 1 |
| Albers 2024 | SoC VS TNK | 1.0522887 | 0.8931215 | 1.239822 | 1 |
| Albers 2024 | SoC VS rt-PA | 1.2245316 | 1.037222 | 1.445668 | 1 |
| Cheng 2025 | SoC VS JX10 | 1.5773727 | 0.9380257 | 2.652491 | 1 |
| Cheng 2025 | SoC VS PBO | 1.0792551 | 0.8987425 | 1.296024 | 1 |
| Cheng 2025 | SoC VS TNK | 1.1682047 | 1.00852 | 1.353174 | 1 |
| Cheng 2025 | SoC VS rt-PA | 1.2256124 | 1.105481 | 1.358799 | 1 |
| Niizuma 2024 | SoC VS PBO | 1.0264064 | 0.8388035 | 1.255968 | 1 |
| Niizuma 2024 | SoC VS TNK | 1.0715894 | 0.9300387 | 1.234684 | 1 |
| Niizuma 2024 | SoC VS rt-PA | 1.2082948 | 1.055253 | 1.383532 | 1 |
| Ringleb 2019 | SoC VS JX10 | 1.470198 | 0.8413247 | 2.569141 | 1 |
| Ringleb 2019 | SoC VS PBO | 1.0059251 | 0.7997316 | 1.265281 | 1 |
| Ringleb 2019 | SoC VS TNK | 1.064217 | 0.9166296 | 1.235568 | 1 |
| Ringleb 2019 | SoC VS rt-PA | 1.2129094 | 1.047277 | 1.404737 | 1 |
| Wang 2023 | SoC VS JX10 | 1.4976703 | 0.8579701 | 2.614329 | 1 |
| Wang 2023 | SoC VS PBO | 1.0247219 | 0.8230892 | 1.275749 | 1 |
| Wang 2023 | SoC VS TNK | 1.0668437 | 0.9057359 | 1.256608 | 1 |
| Wang 2023 | SoC VS rt-PA | 1.2051889 | 1.036339 | 1.401549 | 1 |

Supplementary Table 28.Sensitivity analysis for the 36h Sich outcome in adults with IS

| **dropped_id** | **comparison** | **RR** | **RR lci** | **RR uci** | **connected** |
| --- | --- | --- | --- | --- | --- |
| Ma 2019 | SoC VS JX10 | 0.99597582 | 0.0476426 | 20.82101 | 1 |
| Ma 2019 | SoC VS PBO | 2.7782486 | 0.4109039 | 18.7846 | 1 |
| Ma 2019 | SoC VS TNK | 3.8181806 | 0.8186513 | 17.80795 | 1 |
| Ma 2019 | SoC VS rt-PA | 4.0169852 | 0.8354436 | 19.3145 | 1 |
| Zhou 2025 | disconnected |  |  |  | 0 |
| Yan 2025 | disconnected |  |  |  | 0 |
| Xiong 2024 | SoC VS JX10 | 0.20755855 | 0.0061555 | 6.998743 | 1 |
| Xiong 2024 | SoC VS PBO | 0.57897903 | 0.042772 | 7.837286 | 1 |
| Xiong 2024 | SoC VS TNK | 0.7956978 | 0.0464568 | 13.62847 | 1 |
| Xiong 2024 | SoC VS rt-PA | 4.0169855 | 0.8354441 | 19.31449 | 1 |
| Albers 2024 | SoC VS JX10 | 0.20755858 | 0.0061555 | 6.998747 | 1 |
| Albers 2024 | SoC VS PBO | 0.5789791 | 0.042772 | 7.837292 | 1 |
| Albers 2024 | SoC VS TNK | 3.8181818 | 0.8186513 | 17.80796 | 1 |
| Albers 2024 | SoC VS rt-PA | 4.0169863 | 0.8354441 | 19.31449 | 1 |
| Niizuma 2024 | SoC VS PBO | 1.6050268 | 0.3437281 | 7.494619 | 1 |
| Niizuma 2024 | SoC VS TNK | 2.6740524 | 0.690617 | 10.35387 | 1 |
| Niizuma 2024 | SoC VS rt-PA | 5.8178891 | 1.474805 | 22.95071 | 1 |

Supplementary Table29.Sensitivity analysis for the 90d Death outcome in adults with IS

| **dropped_id** | **comparison** | **RR** | **RR lci** | **RR uci** | **connected** |
| --- | --- | --- | --- | --- | --- |
| Ma 2019 | SoC VS PBO | 0.9565278 | 0.5329897 | 1.716629 | 1 |
| Ma 2019 | SoC VS TNK | 1.0352821 | 0.6697119 | 1.600403 | 1 |
| Ma 2019 | SoC VS rt-PA | 0.87803415 | 0.5312265 | 1.451253 | 1 |
| Zhou 2025 | SoC VS PBO | 0.84353776 | 0.4965517 | 1.432995 | 1 |
| Zhou 2025 | SoC VS TNK | 0.96554413 | 0.6386086 | 1.459854 | 1 |
| Zhou 2025 | SoC VS rt-PA | 0.86814942 | 0.4348801 | 1.733083 | 1 |
| Yan 2025 | SoC VS PBO | 0.90520271 | 0.5480664 | 1.49506 | 1 |
| Yan 2025 | SoC VS TNK | 1.0040863 | 0.6714173 | 1.501584 | 1 |
| Yan 2025 | SoC VS rt-PA | 1.0568456 | 0.6396243 | 1.746217 | 1 |
| Yogendrakumar 2025 | SoC VS PBO | 0.85395615 | 0.5187832 | 1.405676 | 1 |
| Yogendrakumar 2025 | SoC VS TNK | 0.96177104 | 0.6410466 | 1.442958 | 1 |
| Yogendrakumar 2025 | SoC VS rt-PA | 0.93790307 | 0.5997555 | 1.466701 | 1 |
| Xiong 2024 | SoC VS PBO | 0.77408971 | 0.3253723 | 1.841628 | 1 |
| Xiong 2024 | SoC VS TNK | 0.85686172 | 0.3389259 | 2.166291 | 1 |
| Xiong 2024 | SoC VS rt-PA | 0.91136734 | 0.5567523 | 1.491849 | 1 |
| Albers 2024 | SoC VS PBO | 0.68144119 | 0.2690135 | 1.726167 | 1 |
| Albers 2024 | SoC VS TNK | 1.0352823 | 0.669712 | 1.600403 | 1 |
| Albers 2024 | SoC VS rt-PA | 0.87803395 | 0.5312263 | 1.451253 | 1 |


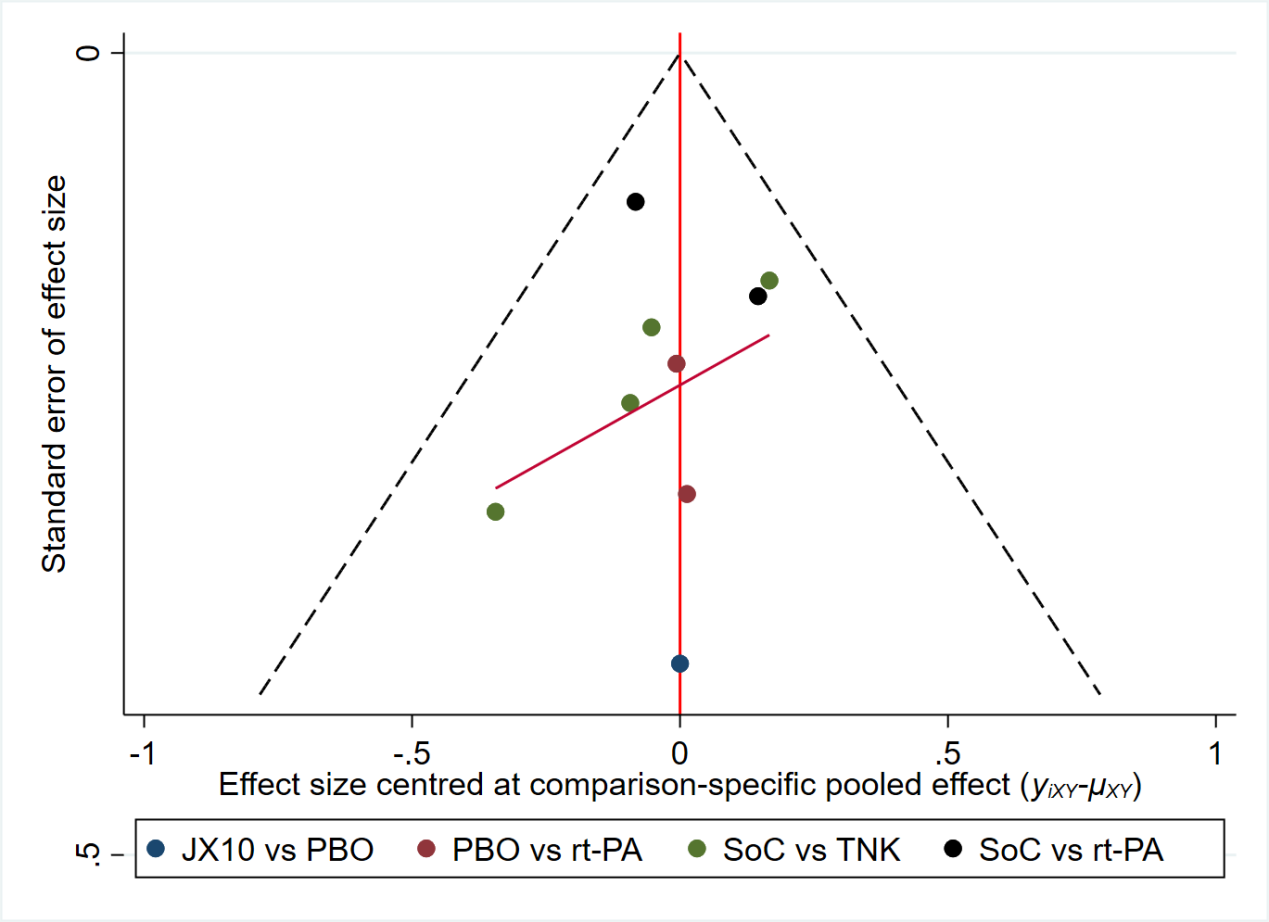


Supplementary Figure 4.Comparison-adjusted funnel plot for the 90d mRS0-1 outcome in adults with IS
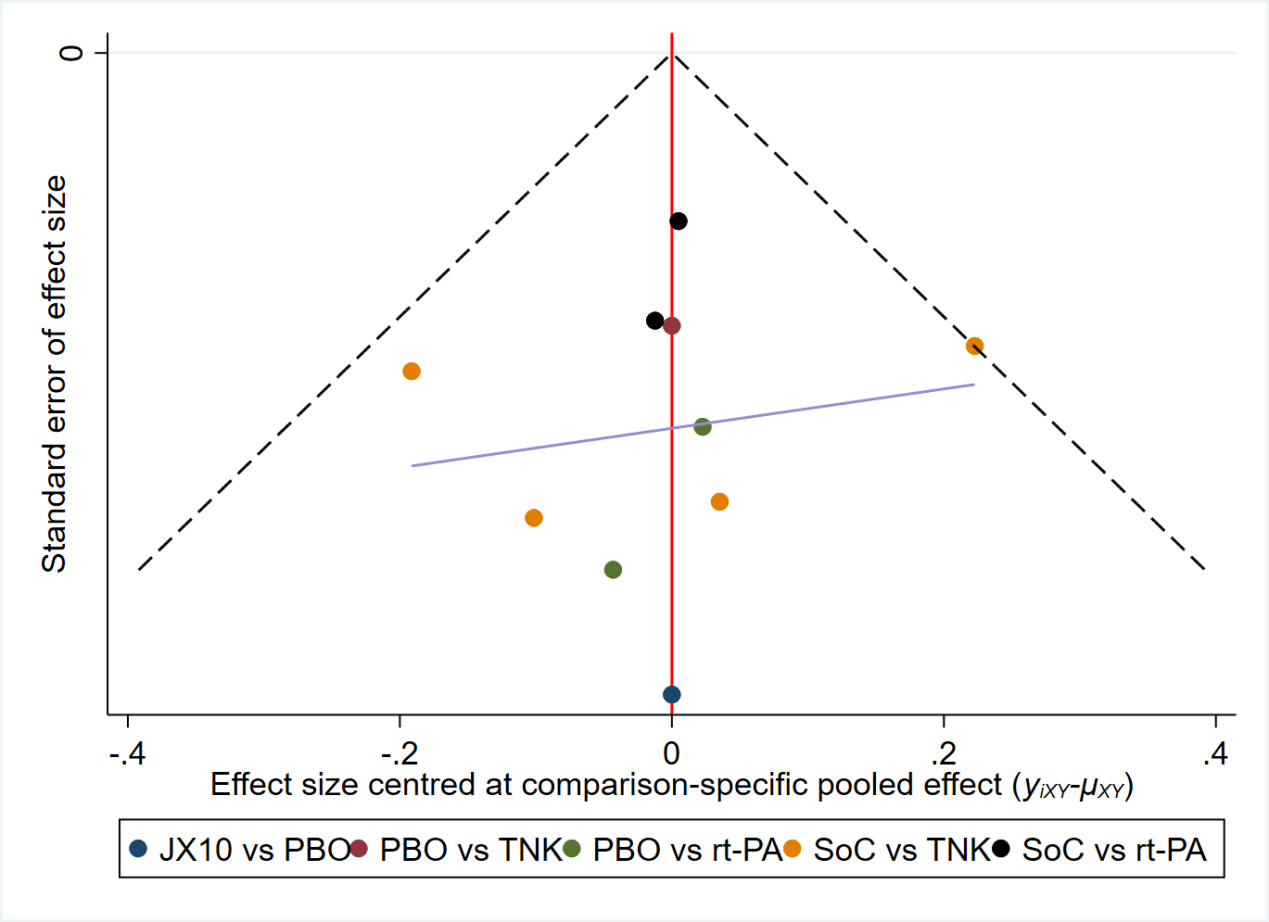
Supplementary Figure 5.Comparison-adjusted funnel plot for the 90d mRS0-2 outcome in adults with IS


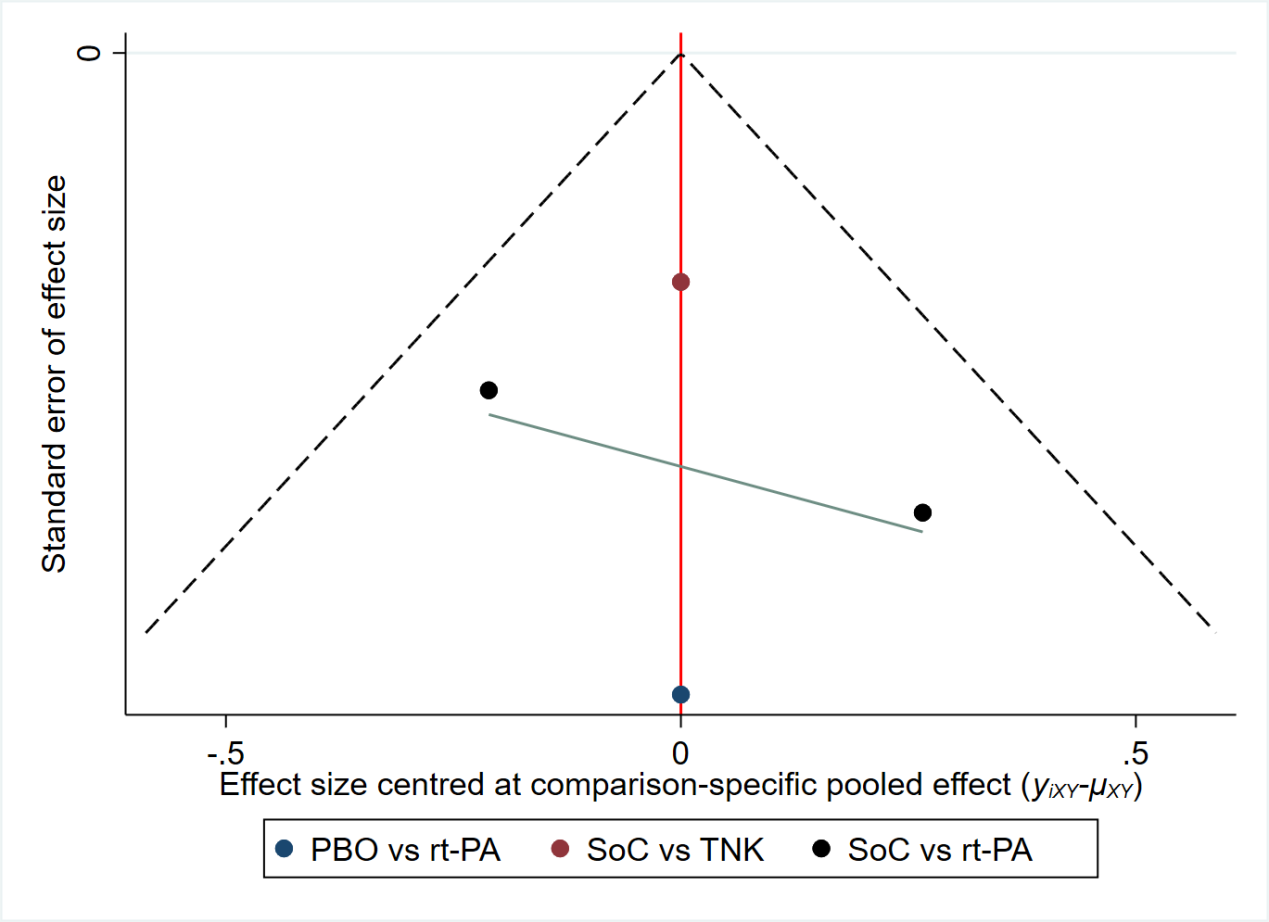


Supplementary Figure 6.Comparison-adjusted funnel plot for the 24h NIHSS outcome in adults with IS


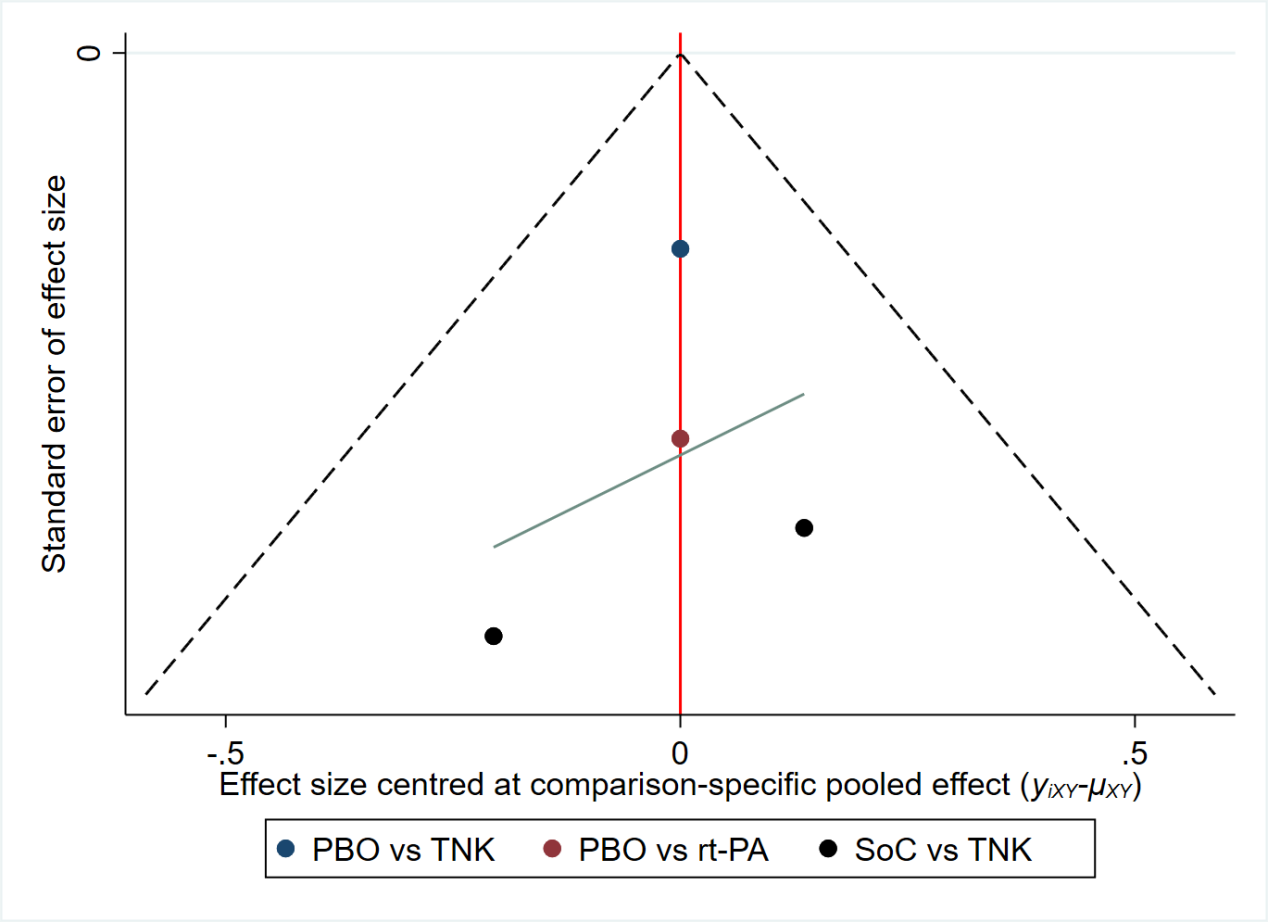


Supplementary Figure 7.Comparison-adjusted funnel plot for the 24h REP outcome in adults with IS


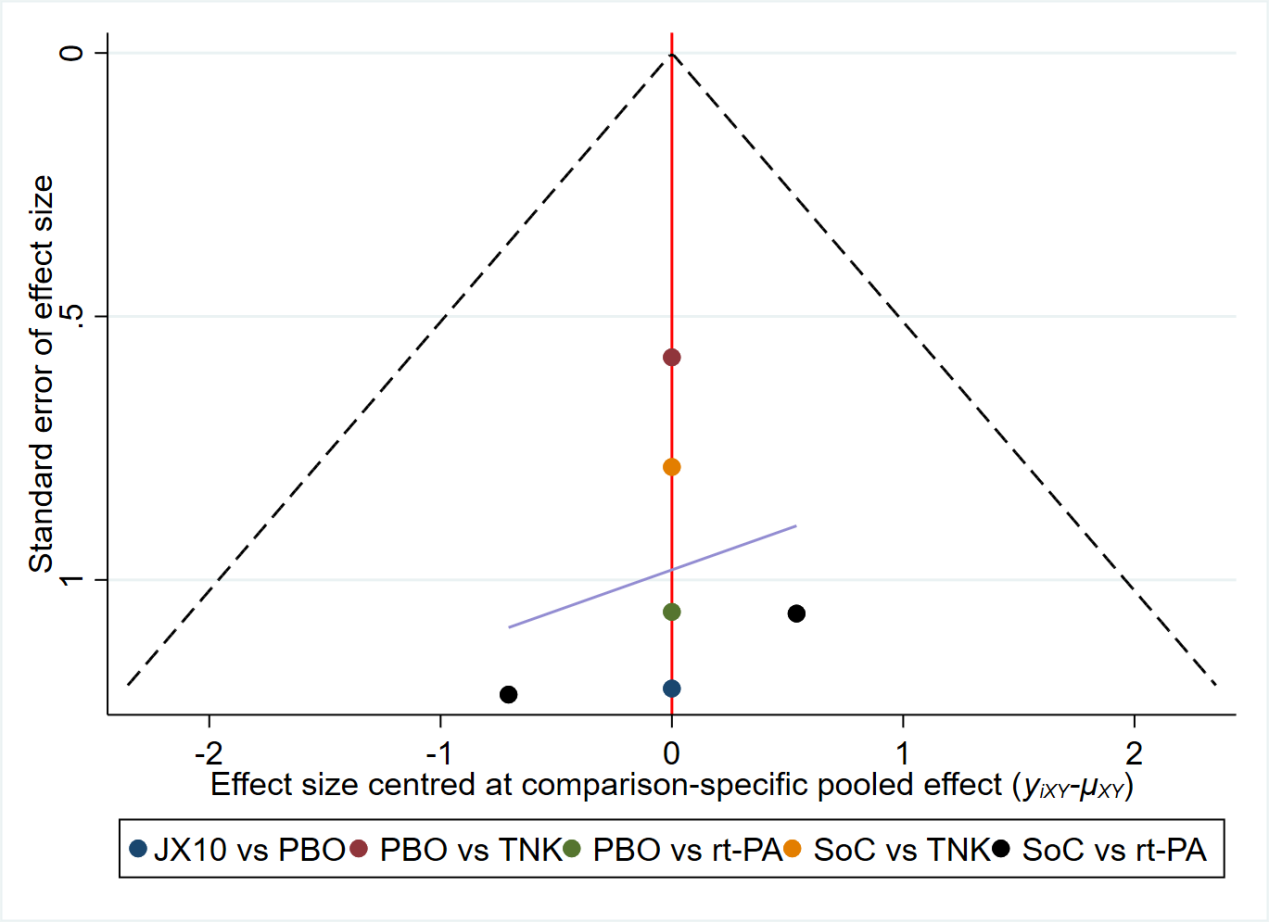


Supplementary Figure 8.Comparison-adjusted funnel plot for the 36h Sich outcome in adults with IS


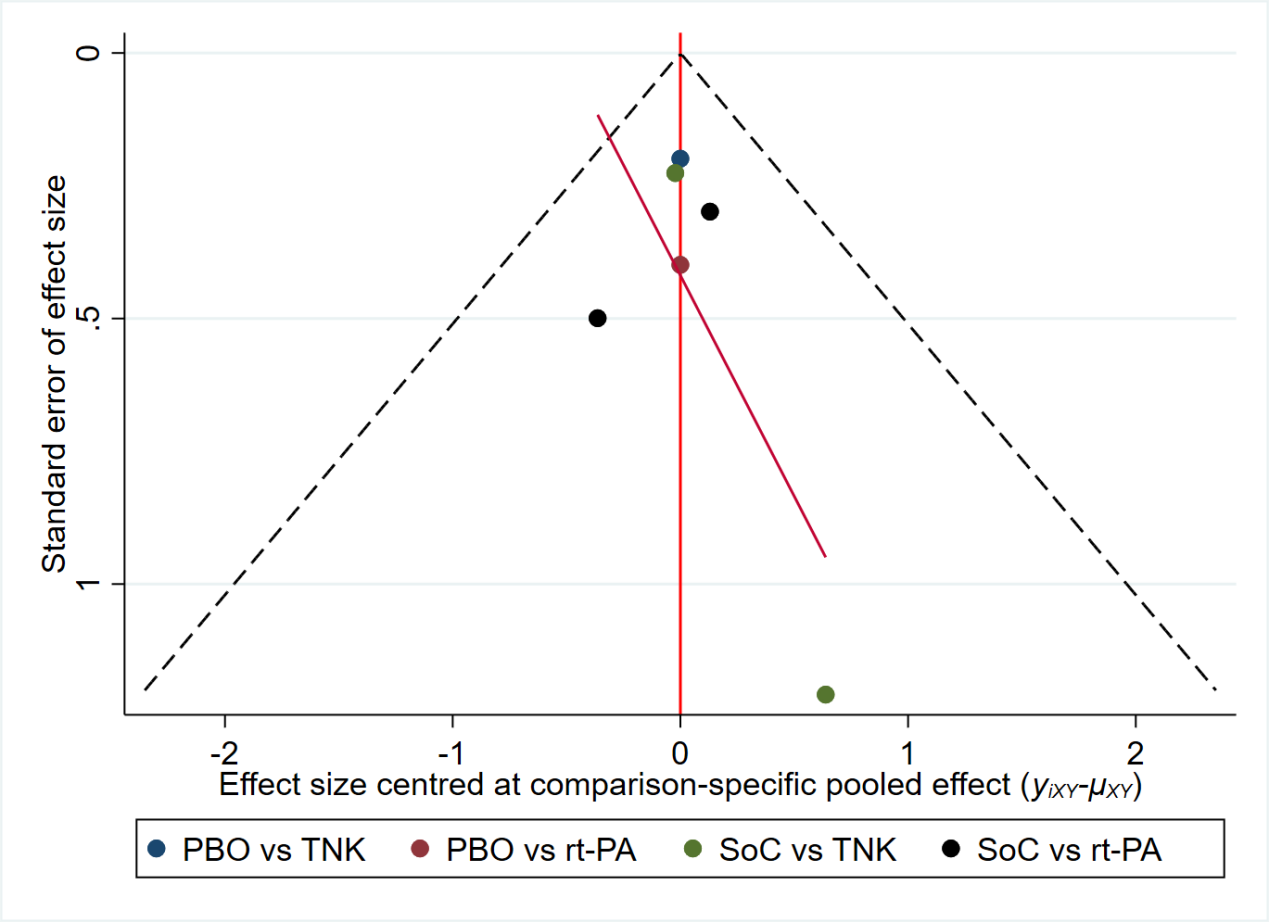


Supplementary Figure 9.Comparison-adjusted funnel plot for the 90d DEATH outcome in adults with IS

Supplementary Table 30.GRADE certainty of evidence for the 90d mRS0-1 outcome in adults with IS

| **Comparison** | **Number of studies** | **Within-study bias** | **Reporting bias** | **Indirectness** | **Imprecision** | **Heterogeneity** | **Incoherence** | **Confidence rating** | **Reason**  **(s) for downgrading** |
| --- | --- | --- | --- | --- | --- | --- | --- | --- | --- |
| JX10:PBO | 1 | Some concerns | Low risk | No concerns | No concerns | Some concerns | No concerns | Low | ["Within-study bias","Heterogeneity"] |
| PBO:rt-PA | 2 | No concerns | Low risk | No concerns | Some concerns | No concerns | No concerns | Moderate | ["Imprecision"] |
| SoC:TNK | 4 | Some concerns | Low risk | No concerns | Some concerns | No concerns | No concerns | Low | ["Within-study bias","Imprecision"] |
| SoC:rt-PA | 2 | Some concerns | Low risk | No concerns | No concerns | No concerns | No concerns | Moderate | ["Within-study bias"] |
| JX10:SoC | 0 | Some concerns | Low risk | No concerns | No concerns | Some concerns | No concerns | Low | ["Within-study bias","Heterogeneity"] |
| JX10:TNK | 0 | Some concerns | Low risk | No concerns | Some concerns | Some concerns | No concerns | Very low | ["Within-study bias","Imprecision","Heterogeneity"] |
| JX10:rt-PA | 0 | Some concerns | Low risk | No concerns | Some concerns | Some concerns | No concerns | Very low | ["Within-study bias","Imprecision","Heterogeneity"] |
| PBO:SoC | 0 | Some concerns | Low risk | No concerns | Some concerns | No concerns | No concerns | Low | ["Within-study bias","Imprecision"] |
| PBO:TNK | 0 | Some concerns | Low risk | No concerns | Some concerns | No concerns | No concerns | Low | ["Within-study bias","Imprecision"] |
| TNK:rt-PA | 0 | Some concerns | Low risk | No concerns | Some concerns | No concerns | No concerns | Low | ["Within-study bias","Imprecision"] |
| JX10:PBO | 1 | Some concerns | Low risk | No concerns | No concerns | Some concerns | No concerns | Low | ["Within-study bias","Heterogeneity"] |
| PBO:rt-PA | 2 | No concerns | Low risk | No concerns | Some concerns | No concerns | No concerns | Moderate | ["Imprecision"] |
| SoC:TNK | 4 | Some concerns | Low risk | No concerns | Some concerns | No concerns | No concerns | Low | ["Within-study bias","Imprecision"] |
| SoC:rt-PA | 2 | Some concerns | Low risk | No concerns | No concerns | No concerns | No concerns | Moderate | ["Within-study bias"] |
| JX10:SoC | 0 | Some concerns | Low risk | No concerns | No concerns | Some concerns | No concerns | Low | ["Within-study bias","Heterogeneity"] |
| JX10:TNK | 0 | Some concerns | Low risk | No concerns | Some concerns | Some concerns | No concerns | Very low | ["Within-study bias","Imprecision","Heterogeneity"] |
| JX10:rt-PA | 0 | Some concerns | Low risk | No concerns | Some concerns | Some concerns | No concerns | Very low | ["Within-study bias","Imprecision","Heterogeneity"] |
| PBO:SoC | 0 | Some concerns | Low risk | No concerns | Some concerns | No concerns | No concerns | Low | ["Within-study bias","Imprecision"] |
| PBO:TNK | 0 | Some concerns | Low risk | No concerns | Some concerns | No concerns | No concerns | Low | ["Within-study bias","Imprecision"] |
| TNK:rt-PA | 0 | Some concerns | Low risk | No concerns | Some concerns | No concerns | No concerns | Low | ["Within-study bias","Imprecision"] |

Supplementary Table 31.GRADE certainty of evidence for the 90d mRS0-2 outcome in adults with IS

| **Comparison** | **Number of studies** | **Within-study bias** | **Reporting bias** | **Indirectness** | **Imprecision** | **Heterogeneity** | **Incoherence** | **Confidence rating** | **Reason**  **(s) for downgrading** |
| --- | --- | --- | --- | --- | --- | --- | --- | --- | --- |
| JX10:PBO | 1 | Some concerns | Low risk | No concerns | Some concerns | Some concerns | No concerns | Very low | ["Within-study bias","Imprecision","Heterogeneity"] |
| PBO:TNK | 1 | Some concerns | Low risk | No concerns | Some concerns | No concerns | No concerns | Low | ["Within-study bias","Imprecision"] |
| PBO:rt-PA | 2 | Some concerns | Low risk | No concerns | Some concerns | No concerns | No concerns | Low | ["Within-study bias","Imprecision"] |
| SoC:TNK | 4 | Some concerns | Low risk | No concerns | No concerns | Some concerns | No concerns | Low | ["Within-study bias","Heterogeneity"] |
| SoC:rt-PA | 2 | Some concerns | Low risk | No concerns | No concerns | Some concerns | No concerns | Low | ["Within-study bias","Heterogeneity"] |
| JX10:SoC | 0 | Some concerns | Low risk | No concerns | Some concerns | Some concerns | No concerns | Very low | ["Within-study bias","Imprecision","Heterogeneity"] |
| JX10:TNK | 0 | Some concerns | Low risk | No concerns | Some concerns | Some concerns | No concerns | Very low | ["Within-study bias","Imprecision","Heterogeneity"] |
| JX10:rt-PA | 0 | Some concerns | Low risk | No concerns | Some concerns | No concerns | No concerns | Low | ["Within-study bias","Imprecision"] |
| PBO:SoC | 0 | Some concerns | Low risk | No concerns | No concerns | Some concerns | No concerns | Low | ["Within-study bias","Heterogeneity"] |
| TNK:rt-PA | 0 | Some concerns | Low risk | No concerns | Some concerns | No concerns | No concerns | Low | ["Within-study bias","Imprecision"] |
| JX10:PBO | 1 | Some concerns | Low risk | No concerns | Some concerns | Some concerns | No concerns | Very low | ["Within-study bias","Imprecision","Heterogeneity"] |
| PBO:TNK | 1 | Some concerns | Low risk | No concerns | Some concerns | No concerns | No concerns | Low | ["Within-study bias","Imprecision"] |
| PBO:rt-PA | 2 | Some concerns | Low risk | No concerns | Some concerns | No concerns | No concerns | Low | ["Within-study bias","Imprecision"] |
| SoC:TNK | 4 | Some concerns | Low risk | No concerns | No concerns | Some concerns | No concerns | Low | ["Within-study bias","Heterogeneity"] |
| SoC:rt-PA | 2 | Some concerns | Low risk | No concerns | No concerns | Some concerns | No concerns | Low | ["Within-study bias","Heterogeneity"] |
| JX10:SoC | 0 | Some concerns | Low risk | No concerns | Some concerns | Some concerns | No concerns | Very low | ["Within-study bias","Imprecision","Heterogeneity"] |
| JX10:TNK | 0 | Some concerns | Low risk | No concerns | Some concerns | Some concerns | No concerns | Very low | ["Within-study bias","Imprecision","Heterogeneity"] |
| JX10:rt-PA | 0 | Some concerns | Low risk | No concerns | Some concerns | No concerns | No concerns | Low | ["Within-study bias","Imprecision"] |
| PBO:SoC | 0 | Some concerns | Low risk | No concerns | No concerns | Some concerns | No concerns | Low | ["Within-study bias","Heterogeneity"] |
| TNK:rt-PA | 0 | Some concerns | Low risk | No concerns | Some concerns | No concerns | No concerns | Low | ["Within-study bias","Imprecision"] |

Supplementary Table 32.GRADE certainty of evidence for the 24h NISS outcome in adults with IS

| **Comparison** | **Number of studies** | **Within-study bias** | **Reporting bias** | **Indirectness** | **Imprecision** | **Heterogeneity** | **Incoherence** | **Confidence rating** | **Reason**  **(s) for downgrading** |
| --- | --- | --- | --- | --- | --- | --- | --- | --- | --- |
| PBO:rt-PA | 1 | No concerns | Low risk | No concerns | No concerns | Some concerns | Some concerns | Low | ["Heterogeneity","Incoherence"] |
| SoC:TNK | 1 | Some concerns | Low risk | No concerns | Some concerns | No concerns | Some concerns | Very low | ["Within-study bias","Imprecision","Incoherence"] |
| rt-PA:SoC | 2 | Some concerns | Low risk | No concerns | Some concerns | No concerns | Some concerns | Very low | ["Within-study bias","Imprecision","Incoherence"] |
| PBO:SoC | 0 | No concerns | Low risk | No concerns | Some concerns | No concerns | Some concerns | Low | ["Imprecision","Incoherence"] |
| PBO:TNK | 0 | Some concerns | Low risk | No concerns | Some concerns | No concerns | Some concerns | Very low | ["Within-study bias","Imprecision","Incoherence"] |
| rt-PA:TNK | 0 | Some concerns | Low risk | No concerns | Some concerns | No concerns | Some concerns | Very low | ["Within-study bias","Imprecision","Incoherence"] |

Supplementary Table 33.GRADE certainty of evidence for the 24h Sich outcome in adults with IS

| **Comparison** | **Number of studies** | **Within-study bias** | **Reporting bias** | **Indirectness** | **Imprecision** | **Heterogeneity** | **Incoherence** | **Confidence rating** | **Reason**  **(s) for downgrading** |
| --- | --- | --- | --- | --- | --- | --- | --- | --- | --- |
| PBO:TNK | 1 | Some concerns | Low risk | No concerns | No concerns | Some concerns | Some concerns | Very low | ["Within-study bias","Heterogeneity","Incoherence"] |
| PBO:rt-PA | 1 | No concerns | Low risk | No concerns | No concerns | Some concerns | Some concerns | Low | ["Heterogeneity","Incoherence"] |
| SoC:TNK | 2 | Some concerns | Low risk | No concerns | No concerns | Some concerns | Some concerns | Very low | ["Within-study bias","Heterogeneity","Incoherence"] |
| PBO:SoC | 0 | Some concerns | Low risk | No concerns | No concerns | Some concerns | Some concerns | Very low | ["Within-study bias","Heterogeneity","Incoherence"] |
| rt-PA:SoC | 0 | Some concerns | Low risk | No concerns | No concerns | Some concerns | Some concerns | Very low | ["Within-study bias","Heterogeneity","Incoherence"] |
| rt-PA:TNK | 0 | No concerns | Low risk | No concerns | No concerns | Some concerns | Some concerns | Low | ["Heterogeneity","Incoherence"] |

Supplementary Table 34.GRADE certainty of evidence for the 36h Rep outcome in adults with IS

| **Comparison** | **Number of studies** | **Within-study bias** | **Reporting bias** | **Indirectness** | **Imprecision** | **Heterogeneity** | **Incoherence** | **Confidence rating** | **Reason**  **(s) for downgrading** |
| --- | --- | --- | --- | --- | --- | --- | --- | --- | --- |
| JX10:PBO | 1 | Some concerns | Low risk | No concerns | Some concerns | No concerns | No concerns | Low | ["Within-study bias","Imprecision"] |
| PBO:TNK | 1 | Some concerns | Low risk | No concerns | Some concerns | No concerns | No concerns | Low | ["Within-study bias","Imprecision"] |
| PBO:rt-PA | 1 | No concerns | Low risk | No concerns | Some concerns | No concerns | No concerns | Moderate | ["Imprecision"] |
| SoC:TNK | 1 | Some concerns | Low risk | No concerns | Some concerns | No concerns | No concerns | Low | ["Within-study bias","Imprecision"] |
| SoC:rt-PA | 2 | Some concerns | Low risk | No concerns | No concerns | Some concerns | No concerns | Low | ["Within-study bias","Heterogeneity"] |
| JX10:SoC | 0 | Some concerns | Low risk | No concerns | Some concerns | No concerns | No concerns | Low | ["Within-study bias","Imprecision"] |
| JX10:TNK | 0 | Some concerns | Low risk | No concerns | Some concerns | No concerns | No concerns | Low | ["Within-study bias","Imprecision"] |
| JX10:rt-PA | 0 | Some concerns | Low risk | No concerns | Some concerns | No concerns | No concerns | Low | ["Within-study bias","Imprecision"] |
| PBO:SoC | 0 | Some concerns | Low risk | No concerns | Some concerns | No concerns | No concerns | Low | ["Within-study bias","Imprecision"] |
| TNK:rt-PA | 0 | Some concerns | Low risk | No concerns | Some concerns | No concerns | No concerns | Low | ["Within-study bias","Imprecision"] |

Supplementary Table 35.GRADE certainty of evidence for the 90d Death outcome in adults with IS

| **Comparison** | **Number of studies** | **Within-study bias** | **Reporting bias** | **Indirectness** | **Imprecision** | **Heterogeneity** | **Incoherence** | **Confidence rating** | **Reason**  **(s) for downgrading** |
| --- | --- | --- | --- | --- | --- | --- | --- | --- | --- |
| PBO:TNK | 1 | Some concerns | Low risk | No concerns | Some concerns | No concerns | No concerns | Low | ["Within-study bias","Imprecision"] |
| PBO:rt-PA | 1 | Some concerns | Low risk | No concerns | Some concerns | No concerns | No concerns | Low | ["Within-study bias","Imprecision"] |
| SoC:TNK | 2 | Some concerns | Low risk | No concerns | Some concerns | No concerns | No concerns | Low | ["Within-study bias","Imprecision"] |
| SoC:rt-PA | 2 | Some concerns | Low risk | No concerns | Some concerns | No concerns | No concerns | Low | ["Within-study bias","Imprecision"] |
| PBO:SoC | 0 | Some concerns | Low risk | No concerns | Some concerns | No concerns | No concerns | Low | ["Within-study bias","Imprecision"] |
| TNK:rt-PA | 0 | Some concerns | Low risk | No concerns | Some concerns | No concerns | No concerns | Low | ["Within-study bias","Imprecision"] |
